# Bromeliaceae subfamilies show divergent trends of genome size evolution

**DOI:** 10.1038/s41598-019-41474-w

**Published:** 2019-03-26

**Authors:** Lilian-Lee B. Müller, Gerhard Zotz, Dirk C. Albach

**Affiliations:** 1 Carl-von-Ossietzky University Oldenburg, Institute of Biology and Environmental Sciences, P.O. Box 2503, 26111 Oldenburg, Germany; 2 Smithsonian Tropical Research Institute, Apartado Postal 0843-03092, Balboa, Ancón, Panamá, Republic of Panama

## Abstract

Genome size is known to vary widely across plants. Yet, the evolutionary drivers and consequences of genome size variation across organisms are far from understood. We investigated genome size variation and evolution in two major subfamilies of the Neotropical family Bromeliaceae by determining new genome size values for 83 species, testing phylogenetic signal in genome size variation, and assessing the fit to different evolutionary models. For a subset of epiphytic bromeliad species, we also evaluated the relationship of genome size with thermal traits and relative growth rate (*RGR*), respectively. Genome size variation in Bromelioideae appears to be evolutionary conserved, while genome size among Tillandsioideae varies considerably, not just due to polyploidy but arguably also due to environmental factors. The subfamilies show fundamental differences in genome size and *RGR*: Bromelioideae have, on average, lower genome sizes than Tillandsioideae and at the same time exhibit higher *RGR*. We attribute this to different resource use strategies in the subfamilies. Analyses among subfamilies, however, revealed unexpected positive relationships between *RGR* and genome size, which might be explained by the nutrient regime during cultivation. Future research should test whether there is indeed a trade-off between genome size and growth efficiency as a function of nutrient supply.

## Introduction

Genome size, i.e., the total amount of nuclear DNA per cell, is known to vary greatly across organisms. For eukaryotes in general, genome sizes differ almost 70 000-fold; genome size in angiosperms still varies >2400-fold^1^. Despite a wealth of studies, the evolutionary drivers and the consequences of such extreme diversity in genome sizes still puzzle scientists. Macro-evolutionary constraints due to past adaptations may partially explain the variability in genome size. However, the fact that genome size can vary considerably even in closely related species^2^ and within species^3,4^ points towards more recent evolutionary events and other mechanisms.

In recent years, it has been demonstrated that the variation in genome size is largely due to varying proportions of non-coding DNA (e.g. tr ansposable elements, satellite DNA, introns)^5,6^. Although several genetic mechanisms, either decreasing or increasing genome size, have been proposed to explain the wide variation in genome size^5,7^, the functional significance of non-coding DNA is still unresolved^8^. Observed correlations between DNA content and cellular traits (e.g., cell size, cell cycle duration) led quite early to the assumption that genome size variation carries functional consequences^9^. Thus, non-coding DNA may play an important role in cellular or physiological processes. A number of recent studies revealed correlations of genome size with cytological, morphological, and physiological traits^10–12^, and, thus, genome size is assumed to influence the range of environmental conditions a plant can tolerate^13^. Knight *et al*.^14^ investigated the cost of carrying a large amount of non-coding DNA and formulated the “large genome constraint hypothesis”: large genomes constrain a species’ evolution, ecology, and phenotype. This might explain why species with very large genomes appear to be excluded from stressful habitats, whereas species with smaller genomes are distributed in widely varying habitats^8,13,15^, which has even been used to explain the evolutionary success of angiosperms as a whole^12^.

**Figure 1:**
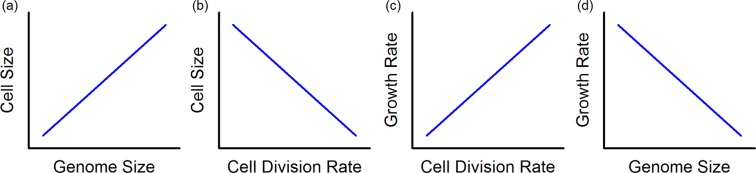
Common relationships between (**a**) genome size and cell size, (**b**) cell division rate and cell size and (**c**) cell division rate and growth rate. Hypothetical resultant relationship between (**d**) genome size and growth rate. Modified after Hessen *et al*.^45^.

On the cellular level an obvious correlate of increased genome size is increased cell size, which in turn is typically associated with reduced cellular metabolic and cell division rate^8,16,17^. Since these traits are directly linked to growth, a negative correlation of genome size and relative growth rate is expected (Fig. 1)^8,11,18^. Besides size-dependent mechanical constraints of metabolic and cell division rates, an evolutionary reallocation of phosphorus (P) from DNA to RNA, i.e., an increase in specific RNA content at the expense of DNA content in order to increase growth rates, has been hypothesised to explain the relationship between genome size and growth rate^18–20^. Moreover, compared to other subcellular structures, genetic material is very rich in nutrients. The proportion of N content by mass in nucleic acids averages 14.5%, the corresponding number for P is 8.7%. This yields a C:N:P ratio of 9.5:3.7:1^20^, which implies increasing demands for N and particular P with increasing genome size. In comparison, the average C:N:P ratio of a chloroplast is 377:80:1^20^. On the tissue level, reported foliar C:nutrient values for vascular plants are even higher, C:N values range from *c*. 5 to >100 and C:P values from <250 to >3500^21^. This illustrates that, in relative terms, it is costly in amounts of N and especially P to build the genome.

It has been hypothesized that species with small genomes are favoured in nutrient-poor environments, due to a reduced demand for nutrients to construct and maintain the genome, allowing them to allocate nutrients elsewhere in support of growth^22^. This hypothesis received experimental support from a study on zooplankton^19^, but has not been explicitly tested in plants, although fertilization experiments in two grassland studies suggested that nutrient availability may play a role in selection of plants with different genome size^23,24^. Further, nutrient limitation might have been a driver of genome size variation in a group of karst plants^25^. Hence, there appears to be an evolutionary pressure towards smaller, more “efficient”, genomes in nutrient-poor environments.

The Neotropics are generally characterized by high micro-environmental heterogeneity and many habitats are nutrient-poor. This exerts strong selective forces and may be one explanation for the remarkably high species richness. One of the largest plant families in the Neotropics are Bromeliaceae, with over 3100 species in 50 genera within eight subfamilies^26^. Their diversity represents an outstanding example of adaptive radiation in plants with a wide range of soil-rooted terrestrial and epiphytic life forms. Characteristic features like absorptive leaf trichomes (i.e., epidermal cells that absorb water and nutrients), phytotelmata (i.e., water-impounding tanks) and the diversification of carbon metabolism allow for an efficient uptake and use of water and nutrients, which in turn allows bromeliads to occur in resource-poor environments^27,28^. Especially in tropical forests, bromeliads are of high ecological importance: they contribute strongly to structural diversity^29^, play a relevant role in forest hydrology and nutrient fluxes^27^ and provide shelter and food for many animals^30^. Few studies have analysed genome sizes in Bromeliaceae^31–34^, which hinders our ability to understand the relevance of genome size variation in bromeliads and its ecological implications. So far, the genome sizes of just *c*. 3% of all bromeliads have been quantified, mostly in the context of taxonomic studies.

Phylogenetic comparative analysis allows the detection of ongoing evolutionary processes and mechanisms driving genome size evolution. To this end, genome sizes have been estimated for *c*. 2% of angiosperm species, covering *c*. 50% of all angiosperm families^35,36^. Unfortunately, the majority of these data are from species from higher latitudes. Thus, the generality of previous findings regarding genome size evolution in plants is biased, since genome sizes of organisms from regions with much higher biodiversity, like the Neotropics, are greatly underrepresented. In studies across a wide array of species, possible contrasting patterns and dynamics of variation in genome size in these regions would be masked.

In this study, we investigated variation of genome size among species within the subfamilies Bromelioideae and Tillandsioideae, the two subfamilies of Bromeliaceae in which most epiphytic species occur and which are known to differ in growth rate^37^, to gain insights into the mechanisms driving genome size evolution. We conducted flow cytometrical measurements to determine DNA contents and present a large data set of new genome size values for 83 bromeliad species. Together with all previously published reports, we examined interspecific variation of DNA content within the subfamilies Bromelioideae and Tillandsioideae, tested phylogenetic signal in genome size and assessed the fit of genome size to different evolutionary models. We also evaluated the relationship of genome size and thermal niche for growth, relative growth rate and growth components, respectively, for a subset of epiphytic bromeliad species.

## Results

### Genome size variation and evolution

**Figure 2:**
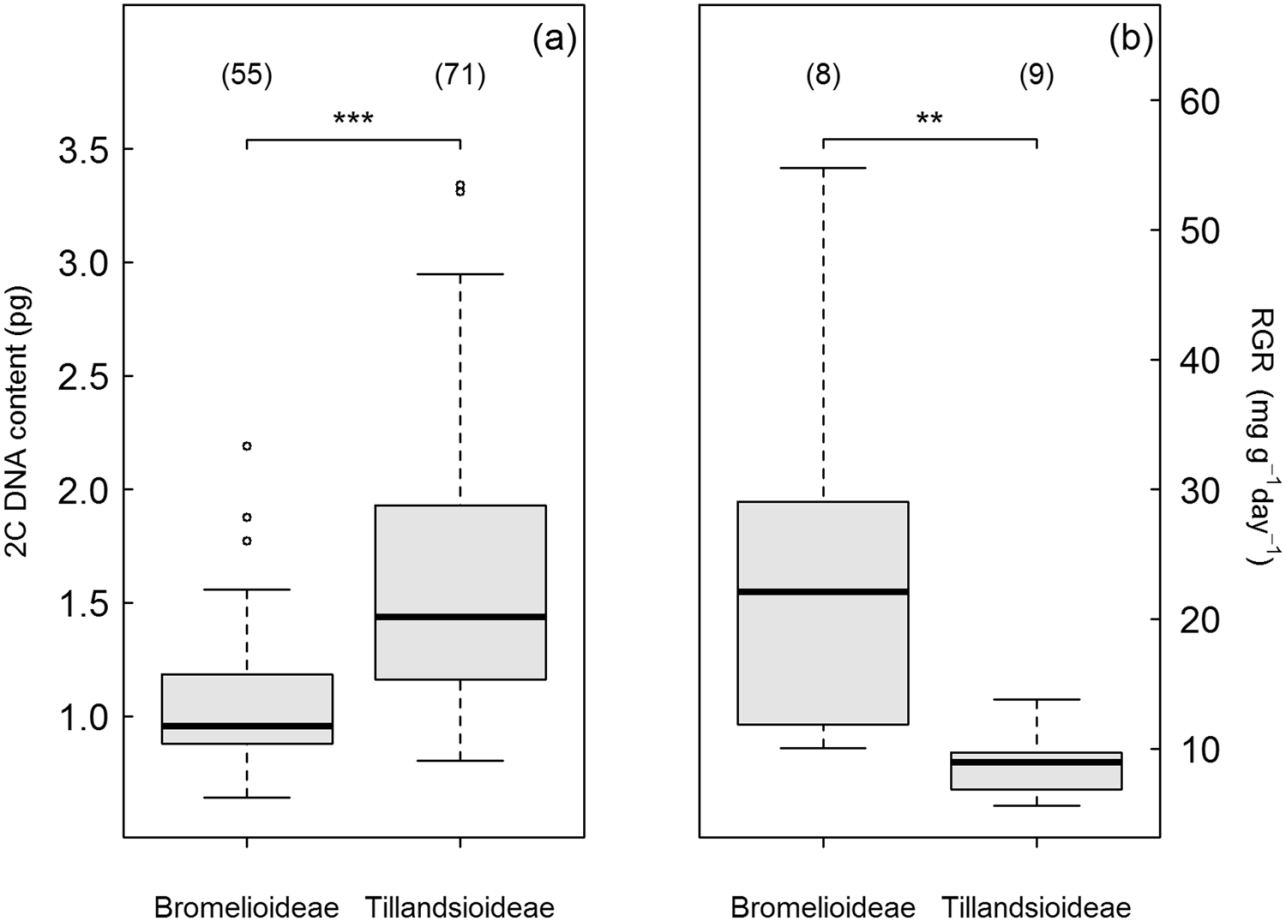
Comparisons of (**a**) genome size (2*C* DNA content) and (**b**) relative growth rate (*RGR*) among bromeliads from the subfamilies Bromelioideae and Tillandsioideae. The median is depicted as bold black bar, the box represents the inner quartile range (IQR), while whiskers extend to extreme values within the 1st Quartile −1.5 × IQR and, respectively, within the 3rd Quartile +1.5 × IQR. Empty circles indicate values below or above this range. *P*-values indicate significant differences between subfamilies (ANOVA/Kruskal-Wallis; **P < 0.01; ***P < 0.001). Species numbers for each group are given in parentheses.

The genome sizes of 89 bromeliad species from the current study, including 83 species that were investigated for the first time, are listed in Supplementary information Table S1. The newly estimated mean 2*C* values varied five-fold, from 0.66 pg in *Billbergia viridiflora* to 3.31 pg in *Tillandsia didisticha*. Both intraspecific variation as well as intra-individual variation (i.e., between runs of the same individual) were <3% among samples. The coefficients of variation (CVs) for G0/G1 peaks of all fresh samples ranged from 1.77 to 5.0, while those from silica-dried material were higher (5.04 to 7.83; Table S1). Combined with values taken from the literature (Table S1), we included genome sizes for 56 of ca. 800 species of Bromelioideae and 71 of ca. 1400 species of Tillandsioideae. Our data on genome size (2*C* DNA content) revealed considerably lower variability in Bromelioideae than in Tillandsioideae (Fig. 2a) and the median genome size of Bromelioideae was >50% smaller than in Tillandsioideae (Kruskal-Wallis: χ^2^ = 42.4, *df* = 1, *P* < 0.001; Fig. 2a).

**Figure 3:**
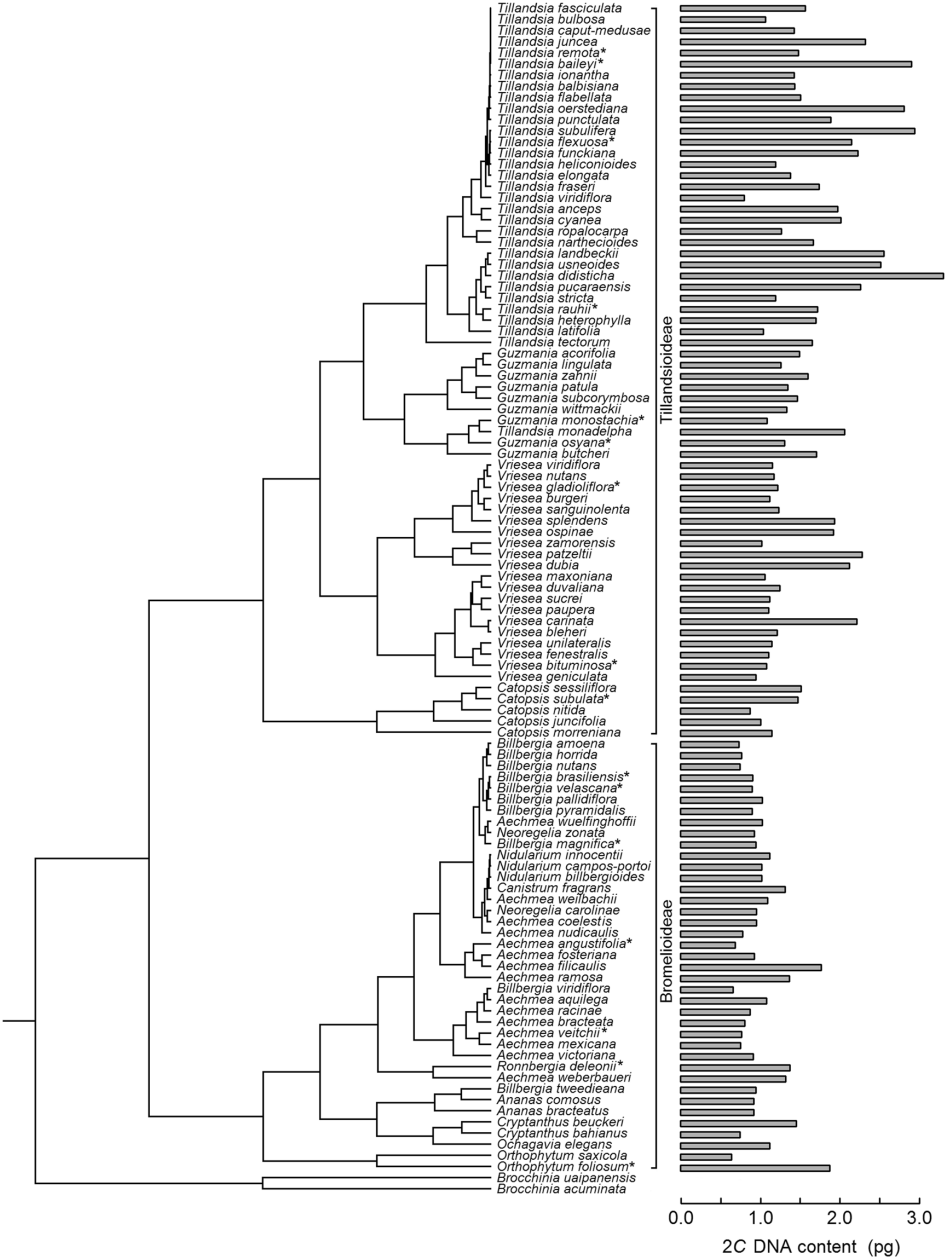
Phylogenetic tree of combined cpDNA dataset (*mat*K, *trn*L-F) of bromeliad species of the subfamilies Tillandsioideae and Bromelioideae based on maximum likelihood, pruned to show only the 105 bromeliad species used in the comparative analysis. *Brocchinia uaipanensis* and *Brocchinia acuminata* are out-groups. Genome size (2*C* DNA content) is mapped to the right of the tree. *Marked species used in the regression analysis.

Both maximum likelihood (ML) and Bayesian phylogenetic analyses based on combined cpDNA datasets of *mat*K and *trn*L-F resulted in trees with similar topologies (Supplementary information Fig. S1), which were generally similar to previously published trees using these markers. Both trees resolved the two subfamilies Bromelioideae and Tillandsioideae, supported by high bootstrap support values (BS; 100 BS) and high posterior probabilities (PP; 1 PP). Furthermore, Tillandsioideae were divided in the strongly supported genera *Catopsis* (100 BS/1 PP), *Vriesea* (75 BS/0.9 PP) and *Tillandsia* plus *Guzmania* (87 BS/1 PP), whereas Bromelioideae did not show a well-supported substructure. The pruned and ultrametricized phylogenetic tree (based on maximum likelihood) of bromeliad species with genome size information for each species is shown in Fig. 3.

Across all species, genome size exhibited a low to moderate phylogenetic signal with Pagel’s λ (λ = 0.31) significantly differing from 0 and 1 (Table 1): genome size (2*C* DNA content) is thus influenced by phylogenetic relationships, i.e. closely related bromeliad species resembled one another more than more distant species. The relatively low estimate of Pagel’s κ (κ = 1.25 × 10^−6^), which is significantly different from 1, but not significantly different from 0 (Table 1), suggests a punctuated mode of genome size evolution in bromeliads. The estimate of Pagel’s δ across all species (δ = 2.99) suggested that genome size evolves according to a model of species-specific adaptation with an accelerated evolution over time but is significantly constrained by the phylogeny (Table 2).

**Table 1:** Likelihood ratio test (LRT) for the observed vs. expected values of phylogenetic scaling parameters for different models of genome size evolution of all bromeliad species, examined in this study and the two bromeliad subfamilies Bromelioideae and Tillandsioideae, separately.

Genome size (*2C*)	Observed value	Log likelihood	*P* for LRT
all species			
Lambda			
**λ estimated**	**0.31**	**−68.36**	
λ forced = 1	—	**−**653.92	<0.001
λ forced = 0	—	**−**86.40	<0.001
Kappa κ			
κ estimated	1.25 × 10^–6^	**−**77.36	
κ forced = 1	—	**−**653.92	<0.001
**κ forced** = **0**	—	**−77.36**	**>0.1**
Delta δ			
**δ estimated**	**2.99**	**−326.62**	
δ forced = 1	—	**−**653.92	<0.001
Bromelioideae			
Lambda			
λ estimated	0.81	**−**4.24	
λ forced = 1	—	**−**8.32	<0.01
**λ forced** = **0**	—	**−5.15**	**>0.1**
Kappa κ			
**κ estimated**	**0.66**	**−6.03**	
κ forced = 1	—	**−**8.32	<0.05
κ forced = 0	—	**−**14.47	<0.001
Delta δ			
**δ estimated**	**2.99**	**−4.96**	
δ forced = 1	—	**−**8.32	<0.01
Tillandsioideae			
Lambda			
**λ estimated**	**0.17**	**−52.39**	
λ forced = 1	—	**−**615.74	<0.001
λ forced = 0	—	**−**54.69	<0.05
Kappa κ			
**κ estimated**	**1.28 × 10** ^**−6**^	**−56.98**	
κ forced = 1	—	**−**655.21	<0.001
κ forced = 0	—	**−**77.69	<0.001
Delta δ			
**δ estimated**	**2.99**	**−275.65**	
δ forced = 1	—	−615.74	<0.001

Observed parameters (λ, κ, δ) were contrasted with values expected under the null hypothesis (values = 0 and 1). When observed models show no significant difference from expectation, the latter was selected. Selected models are indicated in bold.

**Table 2:** Trait evolution model selection statistics for genome size (2*C*) of all bromeliad species, examined in this study and the two bromeliad subfamilies Bromelioideae and Tillandsioideae, separately.

Model	Parameters	Log likelihood	*k*	AICc
all species				
BM		**−**653.92	2	1312.0
**Lambda**	**λ** = **0.31**	**−68.36**	**3**	**143.0**
Kappa	κ = 1.25 × 10^−6^	−77.36	3	161.0
Delta	δ = 2.99	−326.62	3	659.5
OU	α = 6969.87	**−**85.99	3	178.2
Bromelioideae				
BM		**−**8.32	2	21.0
Lambda	λ = 0.81	−4.24	3	15.2
Kappa	κ = 0.66	−6.03	3	18.8
Delta	δ = 2.99	−4.96	3	16.6
**OU**	**α** = **10.04**	**−0.42**	**3**	**7.6**
Tillandsioideae				
BM		**−**615.74	2	1235.7
**Lambda**	**λ** = **0.17**	**−52.39**	**3**	**111.2**
Kappa	κ = 1.28 × 10^−6^	−56.98	3	120.4
Delta	δ = 2.99	−275.65	3	557.7
OU	α = 2226.47	−54.66	3	115.7

Log likelihood, logarithm of the maximized likelihood; *k*, total number of parameter; AICc, second-order estimator of the Akaike information criterion; BM, pure Brownian motion; Lambda (λ), Kappa (κ) and Delta (δ), Pagel’s phylogenetic scaling parameters; OU, Ornstein-Uhlenbeck model. Bold letters indicate the best fitting model.

Separate analyses of the two subfamilies revealed divergent Pagel’s phylogenetic scaling parameters. The estimate of Pagel’s λ across Tillandsioideae (λ = 0.17; Table 1) shows a significant but low phylogenetic signal, which was significantly different from 0 and significantly lower than 1. Across Bromelioideae, however, genome size exhibited a strong phylogenetic signal (λ = 0.81), which was significantly lower than 1, but not significantly different from 0 (Table 1). The estimate of Pagel’s κ across Tillandsioideae was relatively low (κ = 1.28 × 10^–6^) and significantly lower than 1, but not significantly different from 0 (Table 1). By contrast, the evolution of genome size in Bromelioideae (κ = 0.66) is consistent with a gradual mode (increased rates of evolution in shorter branches (0 < κ < 1; Table 1). The relatively high estimates of Pagel’s δ across Tillandsioideae (δ = 2.99), as well as across Bromelioideae (δ = 2.99), which were significantly different from 1 (Table 1), indicate an increasing rate of genome size evolution through time. Maximum likelihood tests of the continuous models revealed that genome size across Tillandsioideae were best fitted by the λ -based model, whereas the OU-based model worked best in the case of Bromelioideae (Table 2).

### Genome size and temperature

**Figure 4:**
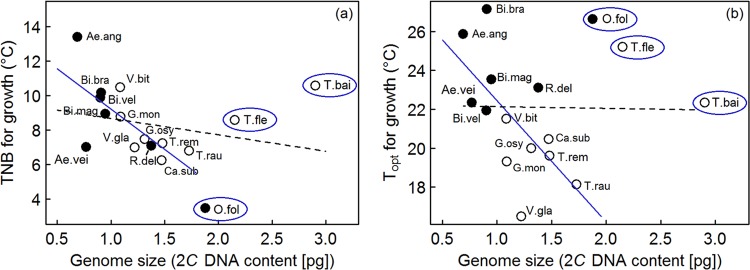
Relationship between genome size (2*C* DNA content) and thermal traits of 16 epiphytic bromeliad species: (**a**) thermal niche breadth (TNB) for growth and (**b**) optimal temperature (T_opt_) for growth across all species (black line) and across species, excluding possible polyploid species (blue line). Full species names are given in Supplementary information Table S1; open dots indicate Tillandsioideae, filled dots Bromelioideae; blue circles label possible polyploid species. Solid and dashed regression lines indicate a significant and non-significant relationship, respectively.

Genome size and thermal niche breadth of 16 epiphytic bromeliad species were unrelated (Pearson Product Moment correlation: *R²* = −0.01, slope = −0.95, *P* > 0.05; Fig. 4a). However, sharp increases in genome size of *Orthophytum foliosum*, *Tillandsia flexuosa* and *T. bailyi* relative to their respective closest related species (see Supplementary Information Figs S2, S3) might indicate polyploidy in these three species. Based on this assumption, we repeated the analysis without these three species. Now, genome size was inversely related to thermal niche breadth (*R²* = 0.46, slope = −0.47, *P* < 0.01; Fig. 4a). There was no significant relationship between genome size and optimal temperature for growth (*R²* = −0.07, slope = −0.09, *P* > 0.05; Fig. 4b), but again, the exclusion of the potentially polyploid species resulted in a significant relationship. Optimal growth temperature decreased with genome size (*R²* = 0.37, slope = −0.63, *P* < 0.05; Fig. 4b).

### Genome size and growth

**Figure 5:**
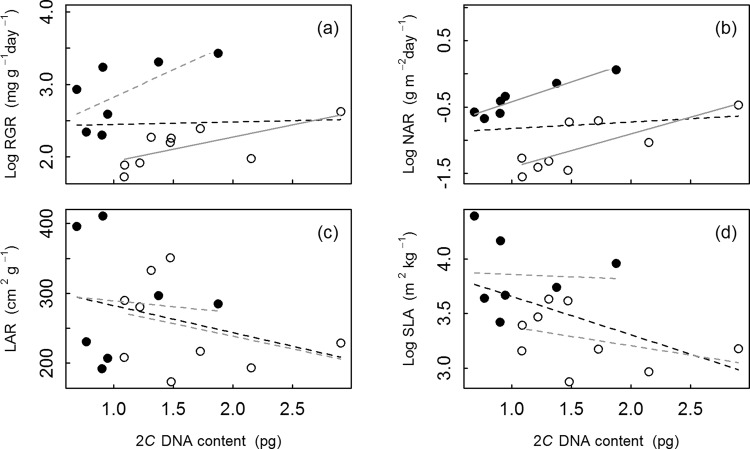
Relationship between genome size (2*C* DNA content) and relative growth rate (*RGR*) and three growth components, respectively, across all bromeliad species (black) and across Bromelioideae and Tillandsioideae separately (grey). (**a**) *RGR*; (**b**) net assimilation rate (*NAR*); (**c**) leaf area ratio (*LAR*) and (**d**) specific leaf area (*SLA*). Data are split into Bromelioideae (closed circles) and Tillandsioideae (open circles). Solid and dashed regression lines indicate a significant and non-significant relationship, respectively.

Genome size was significantly lower in Bromelioideae than in Tillandsioideae (Fig. 2a); the reverse was true for relative growth rate (*RGR;* ANOVA: *F*_1,15_ = 16.0, *P* < 0.01; Fig. 2b). Neither observation can be explained by a potentially larger number of polyploids sampled in Tillandsioideae alone. Across all bromeliad species, the regression analysis showed no relationship between *RGR* and genome size (Pearson Product Moment correlation: *R²* = −0.07, slope = 0.03, *P > *0.1; Table 3; Fig. 5a). At the subfamily level, however, we found a significant positive correlation between *RGR* and genome size across Tillandsioideae (*R²* = 0.41, slope = 0.34, *P* < 0.05) but only a trend across Bromelioideae (*R²* = 0.34, slope = 0.75, *P* < 0.1; Table 3, Fig. 5a). The effect of genome size on *RGR* differed significantly among subfamilies (ANCOVA: F_1,13_ = 31.8; *P* < 0.001). The analysis of the relationship between genome size and the growth components across subfamilies revealed the expected stronger relationship between genome size and NAR, which is almost exclusively due to differences in growth^30^ (Bromelioideae: *R²* = 0.86; slope = 0.60, *P* < 0.001; Tillandsioideae: *R²* = 0.53, slope = 0.50, *P* < 0.05; across all species: *R²* = −0.06, slope = 0.10, *P* > 0.1; Table 3; Fig. 5b). The other growth components LAR and SLA did not show any correlations with genome size (LAR: all species: *R²* = 0.03, slope = −38.78, *P* > 0.1; Bromelioideae: *R²* = −0.19, slope = −16.87, *P* > 0.1; Tillandsioideae: *R²* = −0.01, slope = −36.08; SLA: all species: *R²* = 0.19, slope = −0.35, *P* < 0.1; Bromelioideae: *R²* = −0.20, slope = −0.04, *P* > 0.1; Tillandsioideae: *R²* = 0.02, slope = −0.17, *P* > 0.1; Table 3; Fig. 5d). The effect of genome size on *NAR* differed significantly among subfamilies (ANCOVA: F_1,13_ = 73.5; *P* < 0.001).

**Table 3:** Results of the regression analyses across all bromeliad species (*n* = 16) and for Bromelioideae (*n* = 7) and Tillandsioideae (*n* = 9) analysed individually for genome size (2*C* DNA content) with relative growth rate (*RGR*) and with the growth components net assimilation rate (*NAR*), leaf area ratio (*LAR*) and specific leaf area (*SLA*).

	RGR (mg g^−1^ day^−1^)			NAR (g m^−2^ day^−1^)		
	*R²*	Slope	*P*	*R²*	Slope	*P*
All 2*C* DNA content (pg)	−0.07	0.03	0.892	−0.06	0.10	0.665
Bromelioideae 2*C* DNA content (pg)	0.34	0.75	0.099	0.86	0.60	**0.001**
Tillandsioideae 2*C* DNA content (pg)	0.41	0.34	**0.036**	0.53	0.50	**0.015**
	**LAR (cm² g** ^**−1**^ **)**			**SLA (m² kg** ^**−1**^ **)**		
	***R²***	**Slope**	***P***	***R²***	**Slope**	***P***
All 2*C* DNA content (pg)	0.03	−38.78	0.256	0.19	0.35	0.054
Bromelioideae 2*C* DNA content (pg)	−0.19	−16.87	0.863	0.20	0.04	0.908
Tillandsioideae 2*C* DNA content (pg)	−0.01	−36.08	0.372	0.02	0.17	0.316

Significant relationships (*P* < 0.05) are indicated in bold.

## Discussion

Whereas our knowledge of genome sizes in temperate plants is steadily increasing, our knowledge of tropical plants lags behind. Our work considerably increased the number of known genome sizes in Bromeliaceae by 55% to between five and seven percent of all species for Tillandsioideae and Bromelioideae, respectively. It provides critical information on the ecological basis of genome size evolution. An average genome size of 1.35 pg DNA/2*C* for the studied Bromeliaceae is consistent with the general notion of small genome sizes in tropical plants^38,39^. Genome size variation in the subfamily Tillandsioideae was *c*. 60% higher than in Bromelioideae (absolute 2*C* range of 2.5 pg *vs*. 1.6 pg; *n* = 71 and 56). The observed significant differences in genome size and genome size variation in the two subfamilies confirm and extend the findings of another recent study^32^ but still need to be considered carefully until a larger percentage of species in both subfamilies has been analysed.

Numerous phylogeny-based studies on genome size variation typically demonstrate strong phylogenetic dependency of this trait at various taxonomic levels, from seed plants as a whole, through family, genus and subgenus down to the intraspecific level^2,15,40,41^. However, similar to our analysis of genome size evolution in Bromeliaceae, other recent studies e.g.^42,43^ found contrasting phylogenetic dependency of genome size at different taxonomic levels of the same family or group. This suggests that the phylogenetic signal can vary among different taxonomic scales and highlights the importance of scale. In our study, genome size at the family level displayed a low to moderate phylogenetic signal and followed a λ-based evolutionary model, but this result blurred contrasting results between subfamilies. Whereas genome sizes among Tillandsioideae displayed a low phylogenetic signal and evolved according to a punctual mode, independent of evolutionary time, genome sizes among Bromelioideae showed a strong phylogenetic signal and evolved gradually along the branches of the phylogenetic tree (Table 1). The moderate phylogenetic signal at the family-level arguably results from averaging across subfamilies, i.e., strong in the one and low in the other. Varying phylogenetic dependency of genome size in the two bromeliad clades indicate that it is not as tightly linked among Tillandsioideae, although changes in genome size are tightly linked to phylogenetic relatedness among Bromelioideae. This could indicate that ecological aspects play a more important role in shaping genome size in Tillandsioideae. In this case, genome size divergence even among closely related species may reflect adaptations to different environmental conditions. Such divergence may be repeated across this clade, producing “groups” of distantly related species, convergently adapted to the same ecological conditions^44^. Alternatively, undetected polyploidy could bias phylogenetic analyses. Polyploidy is frequent in Bromeliaceae with about 10% of the species with known chromosome number being polyploid in Bromelioideae and 5% in Tillandsioideae^32^. However, polyploidy may not be the only factor leading to sharp changes in genome sizes, particularly in Tillandsioideae. Potentially, selection pressure for genome downsizing may be so strong in the habitats colonized by diploid and polyploid Tillandsioideae that superfluous DNA is lost quickly from the nucleus, which is indicated by the low value of Pagel’s κ.

In line with the notion of different trajectories of genome size evolution in the two subfamilies, genome size in Bromelioideae seems to have evolved gradually along the branches to a single optimum, indicated by the relatively high estimate of Pagel’s κ and the best fit to a single-optimum OU model. This suggests that changes in genome size in Bromelioideae are likely restricted by some kind of eco-physiological constraint, which pulls genome size towards a lower optimum^45^. Similar gradual changes in genome size have also been detected among birds, mammals and teleost fish in which genome size varies little^46^. In contrast, genome size in Tillandsioideae is more variable and appears to evolve punctuated at branching points, more likely through drastic ecological differentiation than through polyploidy alone, leading to different selection pressure on genome size among species. Punctuated contributions to molecular divergences across angiosperms appear to be common and widespread^47^. A punctual mode of genome size evolution, often caused, but not limited to polyploidization, has been reported in studies utilizing the same statistical approach we used (e.g., Orobanchaceae^48^ or Liliaceae^2^) but the opposite pattern exists^41^. Independent of taxonomic scale, the evolution of genome size in Bromeliaceae seems to be associated with an accelerated tempo (δ => 1), i.e., more diversification of genome size in the recent history of bromeliads compared to that of early branches as found in other taxa e.g.^41^.

Assuming that genome size is indeed linked with niche differentiation, as suggested in previous reviews^13,14,49^, closely related species within Bromelioideae should be more ecologically similar than distantly related species, since we found a strong phylogenetic signal in this clade. In turn, genome size in Bromelioideae might be conserved, potentially constraining genome size variability, as reflected in our results. Currently, our conclusions remain rather hypothetical and, therefore, we suggest that future research should increase taxon sampling, incorporate the necessary ecological data, investigate whether ecological similarity among species is statistically associated with phylogenetic relatedness, and control for ploidy.

One potential ecological factor shaping genome size is temperature. Bromeliads with small genome sizes seem to be able to occupy a broader range of habitats and simultaneously perform optimally under higher temperatures as compared to large genome size species. This may either represent functional relationships between cellular and whole plant physiology and environmental factors or may arise from correlated selection pressures acting directly on genome size. Several previous studies have reported correlations between genome size and ecological variables, such as temperature or elevation, which co-varies with temperature, but others report conflicting results^13,14^. Although genome size appears to be an important trait that may contribute to physiological and climatic differentiation between species, the cause for these correlations is still unresolved^8^. Hence, we can only report these findings, but are currently unable to provide a mechanistic explanation. Nonetheless, if future research validates our findings on genome size and thermal traits within this family, genomes size might be used as an easily measureable trait to assess the vulnerability of these plants to rising temperatures, even without detailed knowledge of the underlying mechanisms.

Regarding the hypothesis that species with small genome grow faster, our study reveals contrasting results. Generally, this idea is based on the assumption that a small genome allows higher allocation of resources to other cellular compartments and hence higher maximum growth rates^8,17^. Since this concept specifically addresses P allocation from DNA to RNA, it is worthwhile to note that P-limitation in bromeliads appears to be strong^50,51^. The significant differences in average relative growth rate^37^ and genome size between the two subfamilies (Fig. 2), which point towards fundamental differences in these traits between Tillandsioideae and Bromelioideae, supports this notion. The analyses among subfamilies, however, revealed positive relationships between *RGR* and genome size (Fig. 5).

A fundamental prerequisite for the expected negative relationship between *RGR* and genome size is that organisms’ growth has to be chronically or at least frequently limited by nutrients, i.e., the allocation of potentially limiting nutrients to either DNA or functional structural components represents a trade-off, which has been hypothesized to be a main evolutionary driver for differences in genome size^19^. Here, our study has an important caveat: The growth rate data set used in our analysis, which has been published elsewhere^37^, is based on nutrient-replete growth conditions, and hence may be biased as compared to natural conditions. Recent studies revealed that under non-limiting nutrient conditions species with large genome size compete successfully with species with smaller genome size in terms of biomass production^19,24^. However, the underlying mechanisms for such a relationship remain unknown. Since genome size is assumed to be positively correlated with cell size^8^, we hypothesize that under nutrient replete conditions a large genome might result in a higher relative RNA content, which in turn should result in higher *RGR*^18,52^ as seen in Fig. 5 for the two subfamilies. If correct, we would expect a positive relationship between *RGR* and genome size across all species, which we did not find. Although this could be caused by the low number of species for which *RGR* is available, we attribute this to fundamental differences in these traits between Tillandsioideae and Bromelioideae, which might be explained by different strategies of nutrient utilization. In contrast to Bromelioideae, which seem to utilize nutrients immediately for growth, Tillandsioideae have been reported to store nutrients (P^50,53^; N^54^). A more efficient and immediate utilization of nutrients in Bromelioideae allows for higher maximum *RGRs*. The “storage strategy” of Tillandsioideae, in contrast, is usually characterized by relatively constant, albeit low, growth rates^55,56^. Hence, species of both subfamilies with similar genome size show remarkable differences in *RGR*.

While several studies have found negative correlations between genome size and *RGR*^11,14^, others have found the opposite relationship^14^, making generalizations difficult. Such inconsistent findings might be a result of the fact that most studies regarding the relationship of genome size to growth do not consider nutrient availability or differences between nutrient regimes across systems. However, the notion that nutrient regimes directly affect the relationship between genome size and ecological traits was recently supported^22^. We only know of a single study that specifically addressed nutrient limitation as a possible driver of genome size evolution in plants^25^. The findings of that study did not fully support the hypothesis of nutrient allocation from DNA to RNA, but suggest a competition for nutrients between DNA synthesis and cellular functions as a possible mechanism for genome size evolution in plant species from nutrient-poor habitats.

## Conclusions

Our findings suggest that different evolutionary processes influence genome size evolution in Bromeliaceae. Whereas genome size variation in Bromelioideae appears to be evolutionarily conserved or at least has a single and low optimum, environmental factors and polyploidy seem to be more heterogeneous factors in shaping genome size among Tillandsioideae. The contrasting results on the next higher taxonomic level highlight the importance to consider different taxonomic scales in phylogenetic analyses. In support of “the large genome constraint hypothesis”^14^, we report relationships between genome size and thermal traits that indicate that large genome species might be constrained in their physiological response to temperature. We, here, did not explore the effect of water relationships as co-variable due to a lack of data on water availability for bromeliads, which could be potentially important due to the known limitations of large genomes in dry environments^57^. Pending further validation, genome size in Bromeliaceae may serve as an easily measurable trait to assess their vulnerability to climate change. Furthermore, we observed fundamental differences in genome size and relative growth rate between subfamilies. We hypothesize that genome size variation in Bromeliaceae is driven by evolutionary pressure towards smaller, more “efficient” genomes in support of growth, possibly due to nutrient limitation. However, further research is needed to test this notion, e.g., by investigating the effect of different nutrient regimes on growth in species differing in genome size.

## Methods

### Plant Material

The genome sizes of 89 bromeliads species (including 83 species which have not been studied previously) out of 11 different genera within the subfamilies Bromelioideae and Tillandsioideae were determined by flow cytometry (species names follow The Plantlist^58^; Table S1). The plants were either cultivated under adequate environmental conditions in the greenhouse or germinated from seeds and grown to seedlings in climate chambers. For some species, material from other botanical gardens were sent to Oldenburg in silica gel and measured within one week after receipt. *Solanum pseudocapsicum* L. (1*C* = 1.295 pg)^59^, *Hedychium gardnerianum* Shepard ex Ker Gawl. (1*C* = 2.01 pg)^60^ and *Solanum lycopersicum* L. ‘Stupicke’ (1*C* = 0.98 pg)^61^ were cultivated and used as internal standards.

### Genome size estimation

For nuclei isolation, approximately 1 cm² of leaf material of each individual of the target species were co-chopped with the same amounts of an internal standard (Table S1) into a homogenous mass by using a razor blade in a petri dish, containing 550 µl nuclei extraction buffer (OTTO I)^62^. After further addition of 550 µl OTTO I buffer, the cell suspension was filtered through a 30 µm CellTric filter (Partec GmbH, Münster, Germany) into a plastic tube, and 50 µl RNase (5% Ribonuclease) were added. After incubation in a water bath for 30 min at 37 °C, 450 µl of the cell suspension were transferred to another plastic tube to which 2 ml 6% propidium iodide (PI)-staining solution containing 0.4 M sodium hydrogen phosphate were added. Nuclei staining were carried out in the dark for at least one hour at 4 °C.

Measurements were performed using a CyFlow SL flow cytometer (Partec GmbH, Münster, Germany) equipped with a green laser (532 nm, 30 mW) as the excitation light source. For most species, three technical replicates of 5000 particles of each individual were studied. Since mainly greenhouse or botanical garden material were used, for some species only one individual was sampled, however, if available different individuals were sampled (Table S1). The mean 2*C*-value of each sample was calculated according to equation 1:

1 $$2C\,{{\rm{value}}}_{{\rm{sample}}}=\frac{{{\rm{mean}}}_{{\rm{sample}}{G}_{0}/{G}_{1}{\rm{peak}}{\rm{value}}}\times 2C\,{\rm{DNA}}\,{{\rm{content}}}_{standard}}{{{\rm{mean}}}_{standard{G}_{0}/{G}_{1}{\rm{peak}}{\rm{value}}}}$$

Only measurements with coefficients of variation (CVs) <5% were considered. However, for some species (mainly silica gel samples) measurements with CVs of 5–8% were also included. In total, the genome size of 143 plant individuals were measured, representing 89 species within the subfamilies Bromelioideae and Tillandsioideae. For later analyses, additional genome size data of 39 species were extracted from publications (Table S1). In total, 2*C*-values are given for 128 species, 83 of which have not been studied previously (Table S1).

### Phylogenetic reconstruction

A phylogenetic tree of 133 bromeliad taxa from the subfamilies Bromelioideae, Tillandsioideae and Brocchinioideae (out-group) was reconstructed based on the two chloroplast DNA regions (cpDNA) *mat*K and *trn*L-F using the data set of a previous study^63^ plus 42 sequences of 21 additional species obtained from GenBank (see Supplementary information Table S2 for GenBank accession numbers). Phylogenetic relationships were reconstructed using the same approach as described elsewhere^63^.

### Phylogenetic-based comparative analysis

All phylogenetic-based comparative analyses were performed using R^64^. The best ML tree was pruned using the R package GEIGER^65^ to include only species for which 2*C* values were available. Polyploidy occurs in about 5–10% of the species in Bromeliaceae^32^ and detectable based on sharp increases in genome size between sister groups. We have detected such increases in few species only (Fig. S3). Analyses were, therefore, conducted disregarding polyploidy but the effect of polyploidy on the results has been considered afterwards in the discussion. In order to limit biases in rapidly evolving lineages the pruned ML phylogram (branch lengths are proportional to change) was ultrametricized (rate smoothed). First, the age of the whole tree was set to 1 using the function “makeChronosCalib” in the R package APE^66^ because no calibration point was available. Second, the relative chronogram was estimated using Penalized Likelihood and Maximum Likelihood with a relaxed clock model to account for heterogeneity among branches^67^. A cross-validation was performed to determine an optimal level of smoothing using the function “chronos” in the R package APE^66^. The best resulting ultrametric tree corresponds to the lambda smoothing parameter of 0. Pagel’s lambda (λ), kappa (κ) and delta (δ) were estimated for species means of genome size (2*C*) using the R package GEIGER^65^ to determine phylogenetic association, mode, and tempo of trait evolution^68^. We preferred Pagel’s lambda over Blomberg’s K based on concerns that Blomberg’s K is influenced considerably by branch lengths uncertainties^69^ and the ability to compare values across studies. A value of λ = 0 indicates that traits are independent from their phylogenetic relationships, while values of λ = 1 suggest the reverse. Intermediate values of 0 < λ < 1 indicate different degrees of phylogenetic signal. A value of the branch length scaling parameter κ of 0 indicates that trait evolution is independent of branch length and therefore a punctuated mode of evolution occurs, whereas κ = 1 suggest trait evolution directly proportional to branch length. Values of κ > 1 indicates proportionally more evolution in longer branches (gradual mode), while κ < 1 suggests proportionally more evolution in shorter branches. To detect differential rates of evolution over time, δ was determined. A value of the path length scaling parameter δ = 1 indicates a gradual (constant) evolution over time. Values of δ < 1 suggest temporally early trait evolution, for example as in adaptive radiations, whereas values of δ > 1 indicate longer paths, which have contributed to trait evolution and suggest accelerated evolution over time. The most appropriate models were determined based on Likelihood ratio test^70^. The best fitting model of trait evolution was determined, by comparing Brownian motion, Pagel’s models (λ, κ, and δ) and Ornstein-Uhlenbeck using estimated log likelihood values and corrected Akaike information criterion (AICc)^71^, using the R package GEIGER^65^. For visualization, bar plots of thermal traits were mapped at the side of the pruned phylogenetic tree using the R package PHYTOOLS^72^.

### Relationship of genome size and temperature

To assess whether genome size might constrain species’ response to climatic parameters, such as temperature, we investigated a possible relationship between genome size and two thermal traits, obtained from elsewhere^37^. For a subset of 16 epiphytic bromeliad species, estimates of genome size (2*C* DNA content) were related by linear regression analysis to estimates of thermal niche breadth for growth and optimal growth temperature, respectively.

### Relationship of genome size and *RGR*

Differences in genome size and genome size variability within the subfamilies Bromelioideae and Tillandsioideae were investigated by using a one-way analysis of variance (ANOVA, Kruskal-Wallis test). To assess the relationship between genome size (2*C* DNA content) and relative growth rate, we obtained data on maximum growth rate for 16 epiphytic bromeliad species, consisting of seven Bromelioideae and nine Tillandsioideae^37^. Relative growth rate (*RGR*) can be broken down in the components net assimilation rate (*NAR*), leaf area ratio (*LAR*) and specific leaf area (*SLA*) and leaf mass ratio (*LMR*). The underlying growth components are related to *RGR* as shown in equation 2 and 3:

2 $$RGR=NAR\ast LAR,$$

3 $$LAR=SLA\ast LMR,$$

in which *RGR* is the product of *NAR* (increase in plant mass per unit leaf area and unit of time) and *LAR* (leaf area per unit plant mass) and the latter in turn is the product of *SLA* (leaf area per unit leaf mass) and *LMR* (fraction of total plant mass allocated to leaves). Since the growth rate components are closer to the cellular level, we also explored a potentially stronger relationship between genome size and growth rate components for the same set of species. The analyses were performed using simple linear regression with genome size as the dependent variable. When necessary, data were log transformed to assure the normality assumption of linear regressions. Analyses were conducted with and without putative polyploids. However, since results did not differ much (see Table S3 and Fig. S4 in Supplementary information), we only present those including all samples. As the data set includes species of the two subfamilies Bromelioideae and Tillandsioideae the influence that each group had on the overall relationship was also investigated. Additionally, a one-way analysis of covariance (ANCOVA) was conducted to determine a possible subfamily-related difference on the impact of genome size on *RGR*. Being aware of the restricted data set of relative growth rates, we forgo a phylogenetic independence contrast (PIC) analysis. All statistical analyses were performed in R^64^.

## Supplementary information

SupplementaryInformation_Mueller et al_Bromeliaceae subfamilies show divergent trends of genome size evolution

## Data Availability

All used DNA sequences are available from GenBank (http://www.ncbi.nlm.nih.gov/genbank/); for Accession Numbers, see Table S2 in Supplementary information.

## References

[1] Pellicer J, Hidalgo O, Dodsworth S, Leitch IJ (2018). Genome Size Diversity and Its Impact on the Evolution of Land Plants. Genes.

[2] Leitch IJ (2007). Punctuated genome size evolution in Liliaceae. J Evolution Biol..

[3] Spinnler F, Stöcklin J (2018). DNA-content and chromosome number in populations of *Poa alpina* in the Alps reflect land use history. Flora.

[4] Bilinski P (2018). Parallel altitudinal clines reveal trends in adaptive evolution of genome size in *Zea mays*. Plos Genetics.

[5] Bennett, M. D. & Leitch, I. J. Genome size evolution in plants In *The evolution of the genome* (ed. Gregory, T. R.) 89–162 (Elsevier, 2005).

[6] Grover, C. E. *et al*. Insights into the evolution of the New World diploid cottons (*Gossypium*, subgenus *Houzingenia*) based on genome sequencing. *Genome Biology and Evolution, evy256*, 10.1093/gbe/evy256 (2018). 10.1093/gbe/evy256 PMC6320677 30476109

[7] Bennetzen JL, Ma J, Devos KM (2005). Mechanisms of recent genome size variation in flowering plants. Ann. Bot..

[8] Doyle JJ, Coate JE (2019). Polyploidy, the Nucleotype, and Novelty: The Impact of Genome Doubling on the Biology of the Cell. International Journal of Plant Sciences.

[9] Bennett, M. D. Nuclear DNA content and minimum generation time in herbaceous plants. *Proc. R. Soc. Lond*., *B, Biol. Sci*. **181**, 109–135 (1972). 10.1098/rspb.1972.0042 4403285

[10] Beaulieu JM, Leitch IJ, Knight CA (2007). Genome size evolution in relation to leaf strategy and metabolic rates revisited. Ann. Bot..

[11] Grotkopp E, Rejmánek M, Sanderson MJ, Rost TL (2004). Evolution of genome size in pines (*Pinus*) and its life-history correlates: supertree analyses. Evolution.

[12] Simonin KA, Roddy AB (2018). Genome downsizing, physiological novelty, and the global dominance of flowering plants. PLoS Biol..

[13] Knight CA, Ackerly DD (2002). Variation in nuclear DNA content across environmental gradients: a quantile regression analysis. Ecol. Lett..

[14] Knight CA, Molinari NA, Petrov DA (2005). The large genome constraint hypothesis: evolution, ecology and phenotype. Ann. Bot..

[15] Kang M (2014). Adaptive and nonadaptive genome size evolution in karst endemic flora of China. New Phytol..

[16] Francis D, Davies MS, Barlow PW (2008). A strong nucleotypic effect on the cell cycle regardless of ploidy level. Ann. Bot..

[17] Šímová I, Herben T (2012). Geometrical constraints in the scaling relationships between genome size, cell size and cell cycle length in herbaceous plants. Proc. Roy. Soc. London, ser. B.

[18] Hessen DO, Jeyasingh PD, Neiman M, Weider LJ (2010). Genome streamlining and the elemental cost of growth. Trends Ecol. Evol..

[19] Hessen DO, Ventura M, Elser JJ (2008). Do phosphorus requirements for RNA limit genome size in crustacean zooplankton?. Genome.

[20] Sterner, R. W. & Elser, J. J. *Ecological stoichiometry: the biology of elements from molecules to the biosphere*. (Princeton University Press, 2002).

[21] Elser J, Fagan W, Kerkhoff A, Swenson N, Enquist B (2010). Biological stoichiometry of plant production: metabolism, scaling and ecological response to global change. New Phytol..

[22] Guignard MS (2017). Impacts of nitrogen and phosphorus: from genomes to natural ecosystems and agriculture. Front. Ecol. Evol..

[23] Šmarda P (2013). Effect of phosphorus availability on the selection of species with different ploidy levels and genome sizes in a long‐term grassland fertilization experiment. New Phytol..

[24] Guignard MS (2016). Genome size and ploidy influence angiosperm species’ biomass under nitrogen and phosphorus limitation. New Phytol..

[25] Kang M, Wang J, Huang H (2015). Nitrogen limitation as a driver of genome size evolution in a group of karst plants. Sci. Rep..

[26] Givnish TJ (2011). Phylogeny, adaptive radiation, and historical biogeography in Bromeliaceae: insights from an eight-locus plastid phylogeny. Am. J. Bot..

[27] Benzing, D. H. *Bromeliaceae: profile of an adaptive radiation*. (Cambridge University Press, 2000).

[28] Givnish TJ (2014). Adaptive radiation, correlated and contingent evolution, and net species diversification in Bromeliaceae. Mol. Phylogenet. Evol..

[29] Kelly DL, Tanner EVJ, Lughadha EMN, Kapos V (1994). Floristics and biogeography of a rain forest in the Venezuelan Andes. J. Biogeogr..

[30] Richardson BA (1999). The bromeliad microcosm and the assessment of faunal diversity in a Neotropical forest. Biotropica.

[31] Moura MN, Forzza RC, Cristiano MP (2018). Reconstruction of ancestral genome size in Pitcairnioideae (Bromeliaceae): what can genome size tell us about the evolutionary history of its five genera?. Bot. J. Linn. Soc..

[32] Gitaí J, Paule J, Zizka G, Schulte K, Benko‐Iseppon AM (2014). Chromosome numbers and DNA content in Bromeliaceae: additional data and critical review. Bot. J. Linn. Soc..

[33] Favoreto FC, Carvalho CR, Lima ABP, Ferreira A, Clarindo WR (2012). Genome size and base composition of Bromeliaceae species assessed by flow cytometry. Plant Syst. Evol..

[34] Ramírez-Morillo IM, Brown GK (2001). The origin of the low chromosome number in *Cryptanthus* (Bromeliaceae). Syst. Bot..

[35] APG III An update of the Angiosperm Phylogeny Group classification for the orders and families of flowering plants: APG III. *Bot. J. Linn. Soc*. **161**, 105–121 (2009).

[36] Bennett, M. D. & Leitch, I. J. *Angiosperm DNA C-values database (release 8.0, Dec. 2012)*, http://www.kew.org/cvalues/ (2012).

[37] Müller L-LB, Albach DC, Zotz G (2018). Growth responses to elevated temperatures and the importance of ontogenetic niche shifts in Bromeliaceae. New Phytol..

[38] Levin D, Funderburg S (1979). Genome size in angiosperms: temperate versus tropical species. Am. Nat..

[39] Chase MW, Hanson L, Albert VA, Whitten WM, Williams NH (2005). Life history evolution and genome size in subtribe *Oncidiinae* (Orchidaceae). Ann. Bot..

[40] Leitch IJ, Soltis DE, Soltis PS, Bennett MD (2005). Evolution of DNA amounts across land plants (Embryophyta). Ann. Bot..

[41] Zahradníček J, Chrtek J, Ferreira MZ, Krahulcová A, Fehrer J (2018). Genome size variation in the genus *Andryala* (Hieraciinae, Asteraceae). Folia Geobot.

[42] Eilam T (2007). Genome size and genome evolution in diploid *Triticeae* species. Genome.

[43] Dušková E (2010). Genome size correlates with growth form, habitat and phylogeny in the Andean genus *Lasiocephalus* (Asteraceae). Preslia.

[44] Losos JB (2008). Phylogenetic niche conservatism, phylogenetic signal and the relationship between phylogenetic relatedness and ecological similarity among species. Ecol. Lett..

[45] Hansen TF (1997). Stabilizing selection and the comparative analysis of adaptation. Evolution.

[46] Kapusta A, Suh A, Feschotte C (2017). Dynamics of genome size evolution in birds and mammals. Proc. Natl. Acad. Sci. USA.

[47] Pagel M, Venditti C, Meade A (2006). Large punctuational contribution of speciation to evolutionary divergence at the molecular level. Science.

[48] Weiss-Schneeweiss H, Greilhuber J, Schneeweiss GM (2006). Genome size evolution in holoparasitic *Orobanche* (Orobanchaceae) and related genera. Am. J. Bot..

[49] Suda J, Meyerson LA, Leitch IJ, Pyšek P (2015). The hidden side of plant invasions: the role of genome size. New Phytol..

[50] Zotz G, Asshoff R (2010). Growth in epiphytic bromeliads: response to the relative supply of phosphorus and nitrogen. Plant Biol..

[51] Wanek W, Zotz G (2011). Are vascular epiphytes nitrogen or phosphorus limited? A study of plant ^15^N fractionation and foliar N: P stoichiometry with the tank bromeliad *Vriesea sanguinolenta*. New Phytol..

[52] Elser J (2003). Growth rate - stoichiometry couplings in diverse biota. Ecol. Lett..

[53] Winkler U, Zotz G (2009). Highly efficient uptake of phosphorus in epiphytic bromeliads. Ann. Bot..

[54] Gonçalves AZ, Mercier H, Oliveira RS, Romero GQ (2016). Trade-off between soluble protein production and nutritional storage in Bromeliaceae. Ann. Bot..

[55] Sommer U (1985). Comparison between steady state and non‐steady state competition: experiments with natural phytoplankton. Limnol. Oceanogr..

[56] Grime JP (1977). Evidence for the existence of three primary strategies in plants and its relevance to ecological and evolutionary theory. Am. Nat..

[57] Beaulieu JM, Leitch IJ, Patel S, Pendharkar A, Knight CA (2008). Genome size is a strong predictor of cell size and stomatal density in angiosperms. New Phytol..

[58] The Plant List. *The Plant List*. Version 1.1. Published on the Internet, http://www.theplantlist.org/ (2013).

[59] Temsch EM, Temsch W, Ehrendorfer-Schratt L, Greilhuber J (2010). Heavy metal pollution, selection, and genome size: the species of the Žerjav study revisited with flow cytometry. J. Bot..

[60] Meudt HM (2015). Is genome downsizing associated with diversification in polyploid lineages of *Veronica*?. Bot. J. Linn. Soc..

[61] Doležel J (1998). Plant genome size estimation by flow cytometry: inter-laboratory comparison. Ann. Bot..

[62] Baranyi M, Greilhuber J (1995). Flow cytometric analysis of genome size variation in cultivated and wild *Pisum sativum* (Fabaceae). Plant Syst. Evol..

[63] Müller L-LB, Albach DC, Zotz G (2017). “Are 3 °C too much?”: thermal niche breadth in Bromeliaceae and global warming. J. Ecol..

[64] R Core Team. *R: a language and environment for statistical computing* (R Foundation for Statistical Computing, Vienna, Austria, 2016).

[65] Harmon LJ, Weir JT, Brock CD, Glor RE, Challenger W (2008). GEIGER: investigating evolutionary radiations. Bioinformatics.

[66] Paradis E, Claude J, Strimmer K (2004). APE: analyses of phylogenetics and evolution in R language. Bioinformatics.

[67] Sanderson MJ (2002). Estimating absolute rates of molecular evolution and divergence times: a penalized likelihood approach. Mol. Biol. Evol..

[68] Pagel M (1999). Inferring the historical patterns of biological evolution. Nature.

[69] Molina-Venegas R, Rodríguez MÁ (2017). Revisiting phylogenetic signal; strong or negligible impacts of polytomies and branch length information?. BMC Evol. Biol..

[70] Huelsenbeck JP, Rannala B (1997). Phylogenetic methods come of age: testing hypotheses in an evolutionary context. Science.

[71] Hurvich CM, Tsai C-L (1989). Regression and time series model selection in small samples. Biometrika.

[72] Revell LJ (2012). Phytools: an R package for phylogenetic comparative biology (and other things). Methods Ecol. Evol..

