# Supplementary material for: Bromeliaceae subfamilies show divergent trends of genome size evolution
Source: Sci Rep. 2019 Mar 26;9:5136. doi: 10.1038/s41598-019-41474-w (PMC6435678; doi:10.1038/s41598-019-41474-w)
Supplement: Supplementary file 1 — SupplementaryInformation_Mueller et al_Bromeliaceae subfamilies show divergent trends of genome size evolution [file 41598_2019_41474_MOESM1_ESM.pdf]

Scientific Reports Supplementary Information

Article title: Bromeliaceae subfamilies show divergent trends of genome size evolution

Authors: Lilian-Lee B. Müller, Gerhard Zotz & Dirk C. Albach

<u>The following Supporting Information is available for this article:</u>

Table S1 List of bromeliad species used in this study and in previous studies with their respective 2*C* DNA content

Table S2 List of taxa used in the phylogenetic analysis with their respective GenBank accession no., accession no. of living collection and or specimen voucher and their respective reference

Table S3 Results of the regression analyses across all bromeliad species and for Bromelioideae and Tillandsioideae analysed individually for genome size with relative growth rate and three growth components, respectively, excluding putative polyploids

Fig. S1 Phylogenetic tree of combined cpDNA dataset for 133 taxa of Bromeliaceae (Bromelioideae and Tillandsioideae) based on maximum likelihood

Fig. S2 Pruned phylogenetic tree of combined cpDNA dataset for107 bromeliad species with respective genome size values shown as colors based on maximum likelihood

Fig. S3 Distribution of genome size among 107 bromeliad species

Fig. S4 Relationship between genome size (2*C* DNA content) and relative growth rate and three growth components, respectively, excluding putative polyploids

Table S1 *List of species used in this study and in previous studies with their respective 2C DNA content.*

Supplementary Information Table S1. List of species used in this study and in previous studies with their respective mean 2*C* DNA content, range of coefficients of variation (CVs), used standard, no. of replicates, no. of runs, used material, accession no. of living collection and or Specimen voucher and their respective reference. — Abbreviations: BGBM= Botanical Garden and Botanical Museum Berlin-Dahlem; BOCH= Botanical Garden of the Ruhr-University Bochum; OLD= Botanical Garden of the Carl von Ossietzky University Oldenburg; REG= Botanical Garden of the University of Regensburg; Hed_gar= *Hedychium gardnerianum* Shepard ex Ker Gawl. (1*C* = 2.01 pg; Meudt *et al.* 2015); Sol_lyc= *Solanum lycopersicum* L. 'Stupicke' (1*C* = 0.98 pg; Doležel *et al.* 1998);Sol_ps= *Solanum pseudocapsicum* L. (1*C* = 1.2946 pg; Temsch *et al.* 2010). — Nomenclature follows The Plant List (2013; http://www.theplantlist.org/).

Species	Mean 2*C* value (pg) ± SD	CV (%) range	Standard	No. rep.	No. runs	Material	Accession no. of living collection or Specimen voucher	Reference
**Bromeliaceae subf. Bromelioideae**								
Brocchinia acuminata L.B.Sm.	0.76 ± –	–	–	–	–	–	–	Ebert & Till 1997
Brocchinia uaipanensis (Maguire) Givnish	0.86 ± –	–	–	–	–	–	–	Ebert & Till 1997
Aechmea angustifolia Poepp. & Endl.	0.69 ± 0.005	2.66−3.72	Sol_ps	3	5	fresh	14-098-06-90 (OLD)	This study
Aechmea aquilega (Salisb.) Griseb.	1.08 ± 0.013	–	–	–		–	–	Gitai *et al.* 2014
Aechmea bracteata (Sw.) Griseb.	0.81 ± 0.005	2.62−3.32	Sol_ps	3	5	fresh	00981 (OLD)	This study
Aechmea coelestis (K.Koch) E.Morren	0.96 ± 0.004	3.26−4.12	Sol_ps	1	3	silica	n.a. (Boch)	This study
Aechmea eurycorymbus Harms	2.19 ± 0.013	–	–	–	–	–	–	Gitai *et al.* 2014
Aechmea fendleri André ex Mez	1.25 ± 0.018	–	–	–	–	–	–	Gitai *et al.* 2014
Aechmea filicaulis (Griseb.) Mez	1.77 ± 0.002	3.17−3.77	Sol_ps	1	3	fresh	14-098-09-90 (OLD)	This study
Aechmea fosteriana L.B.Sm.	0.93 ± 0.001	3.90−4.52	Sol_ps	1	3	fresh	14-098-10-90 (OLD)	This study
Aechmea mexicana Baker	0.76 ± 0.009	3.12−4.45	Sol_ps	3	5	fresh	00961 (OLD)	This study
Aechmea nudicaulis (L.) Griseb.	0.78 ± –	–	–	–	–	–	–	Favoreto *et al.* 2012
Aechmea racinae L.B.Sm	0.88 ± 0.016	4.86−5.04	Sol_ps	1	3	silica	n.a. (Boch)	This study

Species	Mean 2*C* value (pg) ± SD	CV (%) range	Standard	No. rep.	No. runs	Material	Accession no. of living collection or Specimen voucher	Reference
**Bromeliaceae subf. Bromelioideae continue**								
Aechmea ramosa Mart. ex Schult. & Schult.f.	1.37 ± 0.018	–	–	–	–	–	–	Favoreto *et al.* 2012
Aechmea veitchii Baker	0.77 ± 0.008	3.03−3.54	Sol_ps	3	5	fresh	00942 (OLD)	This study
Aechmea victoriana L.B.Sm.	0.92 ± 0.002	2.21−2.49	Sol_ps	1	3	fresh	14-098-07-90 (OLD)	This study
Aechmea weberbauenii Harms	1.32 ± 0.008	2.12−4.30	Sol_ps	1	3	silica	n.a. (Boch)	This study
Aechmea weilbachii Didr.	1.10 ± 0.001	1.97−2.19	Sol_ps	1	3	fresh	14-098-12-90 (OLD)	This study
Aechmea wuelfinghoffii E.Gross	1.03 ± 0.002	2.79−2.97	Sol_ps	1	3	fresh	14-098-13-90 (OLD)	This study
Ananas bracteatus (Lindl.) Schult. & Schult.f.	0.92 ± –	–	–	–	–	–	–	Arumuganathan & Earle 1991
Ananas sagenaria (Arruda) Schult. & Schult.f.	1.00 ± 0.021	–	–	–	–	–	–	Favoreto *et al.* 2012
Ananas comosus (L.) Merr.	0.93 ± 0.003	2.92−3.70	Sol_ps	3	3	fresh	n.a. (OLD)	This study
Billbergia amoena (Lodd.) Lindl.	0.74 ± 0.002	4.74−4.78	Sol_ps	1	3	fresh	14-098-14-90 (OLD)	This study
Billbergia brasiliensis L.B.Sm.	0.91 ± 0.005	2.76−3.95	Sol_ps	3	5	fresh	14-098-16-90 (OLD)	This study
Billbergia euphemiae E.Morren	0.89 ± 0.014	–	–	–	–	–	–	Favoreto *et al.* 2012
Billbergia horrida Regel	0.77 ± 0.005	–	–	–	–	–	–	Favoreto *et al.* 2012
Billbergia magnifica Mez	0.95 ± 0.014	3.31−4.86	Sol_ps	3	5	fresh	14-098-17-90 (OLD)	This study
Billbergia nutans H.Wendl. ex Regel	0.75 ± 0.057	–	–	–	–	–	–	Ramírez-Morillo & Brown 2001
Billbergia pallidiflora Liebm.	1.03 ± 0.009	–	–	–	–	–	–	Gitai *et al.* 2014
Billbergia pyramidalis (Sims) Lindl.	0.90 ± 0.003	2.46−3.09	Sol_ps	1	3	fresh	00933 (OLD)	This study
Billbergia tweedieana Baker	0.95 ± 0.014	–	–	–	–	–	–	Favoreto *et al.* 2012
Billbergia velascana Cárdenas	0.90 ± 0.011	3.18−3.86	Sol_ps	3	5	fresh	14-098-19-90 (OLD)	This study
Billbergia viridiflora H.Wendl.	0.66 ± 0.002	4.22−4.32	Sol_ps	1	3	fresh	00968 (OLD)	This study
Bromelia antiacantha Bertol.	0.81 ± 0.014	–	–	–	–	–	–	Favoreto *et al.* 2012

Species	Mean 2*C* value (pg) ± SD	CV (%) range	Standard	No. rep.	No. runs	Material	Accession no. of living collection or Specimen voucher	Reference
**Bromeliaceae subf. Bromelioideae continue**								
Canistrum fragrans (Linden) Mabb.	1.32 ± 0.010	–	–	–	–	–	–	Gitai *et al.* 2014
Cryptanthus acaulis (Lindl.) Beer	1.38 ± 0.028	–	–	–	–	–	–	Ramírez-Morillo & Brown 2001
Cryptanthus marginatus L.B.Sm.	1.26 ± 0.005	–	–	–	–	–	–	Gitai *et al.* 2014
Cryptanthus bahianus L.B.Sm.	0.75 ± 0.068	–	–	–	–	–	–	Ramírez-Morillo & Brown 2001
Cryptanthus beuckeri E.Morren	1.46 ± 0.018	–	–	–	–	–	–	Ramírez-Morillo & Brown 2001
Cryptanthus praetextus E.Morren ex Baker	1.35 ± 0.014	–	–	–	–	–	–	Gitai *et al.* 2014
Cryptanthus schwackeanus Mez	0.71 ± –	–	–	–	–	–	–	Ramírez-Morillo & Brown 2001
Fascicularia bicolor (Ruiz & Pav.) Mez ssp. *bicolor* E.C.Nelson & Zizka	1.05 ± 0.010	–	–	–	–	–	–	Gitai *et al.* 2014
Fascicularia bicolor (Ruiz & Pav.) Mez ssp. *canaliculata* E.C. Nelson & Zizka	1.12 ± 0.008	–	–	–	–	–	–	Gitai *et al.* 2014
Greigia sphacelata (Ruiz & Pavon) Regel	1.56 ± 0.004	–	–	–	–	–	–	Gitai *et al.* 2014
Neoregelia carolinae (Beer) L.B.Sm.	0.96 ± 0.028	2.34 – 3.35	Sol_ps	3	5	fresh	14-098-30-90 (OLD)	This study
Neoregelia aff. *simulans* L.B.Sm.	0.98 0.004	–	–	–	–	–	–	Favoreto *et al.* 2012
Neoregelia zonata L.B.Sm.	0.93 ± 0.001	2.34 – 2.46	Sol_ps	1	3	fresh	14-098-32-90 (OLD)	This study
Nidularium billbergioides (Schult. & Schult.f.) L.B.Sm.	1.02 ± –	7.16	Sol_ps	1	1	silica	2000/3126 (REG)	This study
Nidularium campos-portoi (L.B.Sm.) Leme	1.03 ± 0.003	2.77 – 3.08	Sol_ps	1	3	fresh	14-098-60-90 (OLD)	This study
Nidularium innocentii Lem.	1.12 ± 0.005	4.26 – 4.73	Sol_ps	1	3	fresh	14-098-33-90 (OLD)	This study
Ochagavia elegans Phil.	1.12 ± 0.015	–	–	–	–	–	–	Gitai *et al.* 2014
Orthophytum foliosum L.B.Sm.	1.88 ± –	7.06	Sol_ps	1	1	silica	336-13-96-84 (BGBM)	This study
Orthophytum saxicola (Ule) L.B.Sm.	0.64 ± 0.014	–	–	–	–	–	–	Ramírez-Morillo & Brown 2001
Portea petropolitana Mez	1.31 ± 0.011	–	–	–	–	–	–	Gitai *et al.* 2014
Pseudananas sagenarius (Arruda) Camargo	1.00 ± 0.021	–	–	–	–	–	–	Favoreto *et al.* 2012

Species	Mean 2*C* value (pg) ± SD	CV (%) range	Standard	No. rep.	No. runs	Material	Accession no. of living collection or Specimen voucher	Reference
**Bromeliaceae subf. Bromelioideae continue**								
Quesnelia arvensis Mez	0.88 ± 0.001	–	–	–	–	–	–	Gitai *et al.* 2014
Ronnbergia deleonii L.B.Sm.	1.38 ± 0.024	3.45−4.70	Sol_ps	3	5	fresh	14-098-35-90 (OLD)	This study
**Bromeliaceae subf. Tillandsioideae**								
Catopsis juncifolia Mez & Wercklé	1.01 ± 0.001	3.04−3.46	Sol_ps	1	3	fresh	00972 (OLD)	This study
Catopsis morreniana Mez	1.15 ± 0.013	–	–	–	–	–	–	Gitai *et al.* 2014
Catopsis nitida (Hook.) Griseb.	0.88 ± 0.011	3.97−4.46	Sol_ps	1	3	fresh	n.a. (OLD)	This study
Catopsis sessiliflora (Ruiz & Pav.) Mez	1.52 ± 0.002	2.78−3.21	Sol_ps	1	3	fresh	n.a. (OLD)	This study
Catopsis subulata L.B.Sm.	1.47 ± 0.001	2.32−2.62	Sol_ps	1	3	fresh	n.a. (OLD)	This study
Guzmania acorifolia (Griseb.) Mez	1.50 ± 0.004	2.73−3.78	Sol_ps	1	3	fresh	00973 (OLD)	This study
Guzmania butcheri Rauh	1.71 ± 0.005	2.50−3.44	Sol_ps	1	3	fresh	n.a. (OLD)	This study
Guzmania lingulata (L.) Mez	1.26 ± 0.003	3.64−3.91	Sol_ps	1	3	fresh	14-098-26-90 (OLD)	This study
Guzmania monostachia (L.) Rusby ex Mez	1.09 ± 0.001	2.40−2.94	Sol_ps	2	4	fresh	00966 (OLD)	This study
Guzmania osyana (E.Morren) Mez	1.31 ± 0.017	2.01−2.99	Sol_ps	3	5	fresh	14-127-04-90 (OLD)	This study
Guzmania patula Mez & Wercklé	1.35 ± 0.002	1.94−2.34	Sol_ps	1	3	fresh	00974 (OLD)	This study
Guzmania subcorymbosa L.B.Sm.	1.47 ± 0.002	1.77−3.93	Sol_ps	2	6	fresh	n.a. (OLD)	This study
Guzmania wittmackii (André) André ex Mez	1.34 ± 0.028	3.02−4.45	Sol_ps	2	6	fresh	00992 (OLD)	This study
Guzmania zahnii (Hook.f.) Mez	1.61 ± –	2.49	Sol_ps	1	1	fresh	14-098-28-90 (OLD)	This study
Tillandsia anceps Lodd.	1.98 ± 0.017	1.99−3.92	Sol_ps	4	8	fresh	00998 (OLD)	This study
Tillandsia baileyi Rose ex Small	2.91 ± 0.003	2.69−3.07	Hed_gar	1	3	fresh	00990 (OLD)	This study
Tillandsia balbisiana Schult. & Schult.f.	1.44 ± 0.002	1.94−2.44	Sol_ps	1	3	fresh	01001 (OLD)	This study
Tillandsia brachycaulos Schltdl.	1.51 ± 0.003	–	–	–	–	–	–	Gitai *et al.* 2014
Tillandsia bulbosa Hook.	1.07 ± 0.002	3.57−4.36	Sol_ps	1	3	fresh	n.a. (OLD)	This study

Species	Mean 2*C* value (pg) ± SD	CV (%) range	Standard	No. rep.	No. runs	Material	Accession no. of living collection or Specimen voucher	Reference
**Bromeliaceae subf. Tillandsioideae continue**								
Tillandsia caput-medusae E.Morren	1.43 ± 0.002	2.56−2.72	Sol_ps	1	3	fresh	00987 (OLD)	This study
Tillandsia cyanea Linden ex K.Koch	2.02 ± 0.013	2.13−3.74	Sol_ps	3	5	fresh	00960 (OLD)	This study
Tillandsia didisticha (E.Morren) Baker	3.31 ± 0.086	3.27−4.45	Hed_gar	2	4	fresh	00977 (OLD)	This study
Tillandsia dyeriana André	1.40 ± 0.012	2.17−2.89	Sol_ps	2	2	fresh	n.a. (OLD)	This study
Tillandsia elongata Kunth	1.38 ± 0.003	2.70−3.29	Sol_ps	1	3	fresh	n.a. (OLD)	This study
Tillandsia fasciculata Sw.	1.57 ± 0.024	2.16−2.65	Sol_ps	3	5	fresh	00952 (OLD)	This study
Tillandsia flabellata Baker	1.51 ± 0.019	2.35−3.40	Sol_ps	3	5	fresh	14-098-40-90 (OLD)	This study
Tillandsia flexuosa Sw.	2.15 ± 0.034	3.22−4.61	Sol_ps	3	5	fresh	n.a. (OLD)	This study
Tillandsia fraseri Baker	1.74 ± −	7.83	Sol_ps	1	1	silica	01275 (OLD)	This study
Tillandsia funckiana Baker	2.23 ± 0.003	4.03−4.95	Sol_ps	1	3	fresh	14-098-41-90 (OLD)	This study
Tillandsia heliconioides (Kunth)	1.20 ± 0.002	2.87−3.55	Sol_ps	2	6	fresh	n.a. (OLD)	This study
Tillandsia heterophylla E.Morren	1.71 ± 0.009	2.78−2.98	Sol_ps	3	5	fresh	00959 (OLD)	This study
Tillandsia ionantha Planch.	1.43 ± 0.000	3.85−4.74	Sol_ps	1	3	fresh	00940 (OLD)	This study
Tillandsia juncea (Ruiz & Pav.) Poir.	2.33 ± 0.006	2.41−2.74	Sol_ps	1	3	fresh	00982 (OLD)	This study
Tillandsia landbeckii Phil.	2.56 ± 0.038	2.34−2.48	Hed_gar	3	3	fresh	00965 (OLD)	This study
Tillandsia latifolia Meyen	1.04 ± 0.020	−	−	−	−	−	−	Gitai *et al.* 2014
Tillandsia loliacea Mart. ex. Schult.f.	3.34 ± 0.028	−	−	−	−	−	−	Favoreto *et al.* 2012
Tillandsia monadelpha (E.Morren) Baker	2.06 ± 0.002	4.32−5.00	Sol_ps	1	3	fresh	n.a. (OLD)	This study
Tillandsia narthecioides C.Presl	1.67 ± 0.034	3.57−5.00	Sol_ps	3	5	fresh	00954 (OLD)	This study
Tillandsia oerstediana L.B.Sm.	2.81 ± 0.003	2.41−2.68	Hed_gar	1	3	fresh	00989 (OLD)	This study
Tillandsia pucaraensis Ehlers	2.26 ± 0.004	3.21−4.06	Sol_ps	1	3	fresh	00956 (OLD)	This study
Tillandsia punctulata Schltdl. & Cham.	1.89 ± 0.008	1.85−4.00	Sol_ps	1	3	silica	n.a. (Boch)	This study

Species	Mean 2*C* value (pg) ± SD	CV (%) range	Standard	No. rep.	No. runs	Material	Accession no. of living collection or Specimen voucher	Reference
**Bromeliaceae subf. Tillandsioideae continue**								
Tillandsia rauhii L.B.Sm.	1.73 ± 0.001	5.35−5.80	Hed_gar	1	3	silica	01273 (OLD)	This study
Tillandsia remota Wittm.	1.48 ± 0.002	2.54−2.96	Sol_ps	1	3	fresh	01000 (OLD)	This study
Tillandsia ropalocarpa André	1.27 ± 0.022	−	−	−	−	−	−	Gitai et al. 2014
Tillandsia stricta Sol. ex Ker Gawl.	1.20 ± 0.000	−	−	−	−	−	−	Favoreto et al. 2012
Tillandsia subulifera Mez	2.95 ± 0.007	2.80−4.15	Sol_lyc	1	3	fresh	n.a. (OLD)	This study
Tillandsia tectorum E.Morren	1.66 ± 0.003	2.74−3.44	Sol_ps	2	4	fresh	00955 (OLD)	This study
Tillandsia usneoides (L.) L.	2.52 ± −	−	−	−	−	−	−	Zonneveld et al. 2005
Tillandsia viridiflora (Beer) Baker	0.80 ± 0.006	2.57−3.50	Sol_ps	3	5	fresh	00948 (OLD)	This study
Vriesea bituminosa Wawra	1.09 ± 0.001	2.08−2.36	Sol_ps	1	3	fresh	00993 (OLD)	This study
Vriesea bleheri Roeth & W.Weber	1.22 ± 0.001	3.27−3.72	Sol_ps	1	3	fresh	14-098-50-90 (OLD)	This study
Vriesea burgeri L.B.Sm.	1.12 ± 0.004	3.09−3.28	Sol_ps	1	3	fresh	n.a. (OLD)	This study
Vriesea carinata Wawra	2.22 ± 0.005	2.42−3.20	Sol_ps	1	4	silica	n.a. (Boch)	This study
Vriesea dubia (L.B.Sm.) L.B.Sm.	2.13 ± 0.004	4.29−4.52	Hed_gar	1	3	fresh	14-098-49-90 (OLD)	This study
Vriesea duvaliana E.Morren	1.25 ± 0.003	2.22−3.07	Sol_ps	3	5	fresh	14-098-51-90 (OLD)	This study
Vriesea fenestralis Linden & André	1.11 ± 0.002	2.33−2.39	Sol_ps	1	3	fresh	14-098-52-90 (OLD)	This study
Vriesea geniculata (Wawra) Wawra	0.95 ± 0.003	2.85−3.33	Sol_ps	1	3	fresh	2007GR01394 (BGU)	This study
Vriesea gladioliflora (H.Wendl.) Antoine	1.22 ± 0.003	2.23−3.35	Sol_ps	3	9	fresh	00991 (OLD)	This study
Vriesea maxoniana (L.B.Sm.) L.B.Sm.	1.06 ± 0.003	2.63−2.70	Sol_ps	1	3	fresh	00963 (OLD)	This study
Vriesea nutans L.B.Sm.	1.18 ± 0.001	2.24−2.45	Sol_ps	1	3	fresh	14-098-58-90 (OLD)	This study
Vriesea ospinae var. *gruberi* H. Luther	1.92 ± 0.005	3.09−3.45	Sol_ps	1	3	fresh	00969 (OLD)	This study
Vriesea patzeltii Rauh	2.29 ± 0.001	2.58−3.65	Sol_ps	1	3	fresh	00995 (OLD)	This study
Vriesea paupera (Mez & Sodiro) L.B.Sm. & Pittendr.	1.11 ± 0.002	3.17−4.00	Sol_ps	1	3	fresh	00994 (OLD)	This study

Species	Mean 2*C* value (pg) ± SD	CV (%) range	Standard	No. rep.	No. runs	Material	Accession no. of living collection or Specimen voucher	Reference
**Bromeliaceae subf. Tillandsioideae continue**								
Vriesea sanguinolenta Cogn. & Marchal	1.24 ± 0.003	2.04−2.55	Sol_ps	3	5	fresh	00947 (OLD)	This study
Vriesea scalaris E. Morren	1.11 ± 0.013	–	–	–	–	–	–	Favoreto *et al.* 2012
Vriesea splendens (Brongn.) Lem.	1.94 ± 0.002	3.33−3.64	Sol_ps	1	3	fresh	n.a. (OLD)	This study
Vriesea sucrei L.B.Sm. & Read	1.12 ± 0.001	2.28−2.46	Sol_ps	1	3	fresh	00938 (OLD)	This study
Vriesea unilateralis (Baker) Mez	1.15 ± 0.002	1.86−2.46	Sol_ps	1	3	fresh	01274 (OLD)	This study
Vriesea viridiflora (Regel) Wittm. ex Mez	1.15 ± 0.001	2.41−3.47	Sol_ps	1	3	fresh	n.a. (OLD)	This study
Vriesea vittata (Mez & Wercklé) L.B.Sm. & Pittendr.	1.17 ± 0.002	2.79−2.95	Sol_ps	1	3	fresh	n.a. (OLD)	This study
Vriesea zamorensis (L.B.Sm.) L.B.Sm.	1.03 ± 0.002	2.68−2.74	Sol_ps	1	3	fresh	00971 (OLD)	This study

References

Arumuganathan, K. & Earle, E. (1991). Nuclear DNA content of some important plant species. *Plant molecular biology reporter*, **9,** 208-218.

Doležel, J., Greilhuber, J., Lucretti, S., Meister, A., Lysák, M., Nardi, L. *et al.* (1998). Plant genome size estimation by flow cytometry: inter-laboratory comparison. *Annals of Botany*, **82,** 17-26.

Ebert, I. & Till, W. (1997). Nuclear genome size in Pitcairnioideae (Bromeliaceae) with emphasis on the genus *Pitcairnia*. In: *Abstracts, angiosperm genome size discussion meeting*, p. 15.

Favoreto, F.C., Carvalho, C.R., Lima, A.B.P., Ferreira, A. & Clarindo, W.R. (2012). Genome size and base composition of Bromeliaceae species assessed by flow cytometry. *Plant Systematics and Evolution*, **298,** 1185-1193.

Gitaí, J., Paule, J., Zizka, G., Schulte, K. & Benko-Iseppon, A.M. (2014). Chromosome numbers and DNA content in Bromeliaceae: additional data and critical review. *Botanical Journal of the Linnean Society*, **176,** 349-368.

Meudt, H.M., Rojas-Andrés, B.M., Prebble, J.M., Low, E., Garnock-Jones, P.J. & Albach, D.C. (2015). Is genome downsizing associated with diversification in polyploid lineages of Veronica? *Botanical Journal of the Linnean Society*, **178,** 243-266.

Ramírez-Morillo, I.M. & Brown, G.K. (2001). The origin of the low chromosome number in *Cryptanthus* (Bromeliaceae). *Systematic Botany*, **26,** 722-726.

Temsch, E.M., Temsch, W., Ehrendorfer-Schratt, L. & Greilhuber, J. (2010). Heavy metal pollution, selection, and genome size: the species of the Žerjav study revisited with flow cytometry. *Journal of Botany*, **2010**.

Zonneveld, B., Leitch, I. & Bennett, M. (2005). First nuclear DNA amounts in more than 300 angiosperms. *Annals of Botany*, **96,** 229-244.

Table S2 *List of taxa used in this study with their respective GenBank accession no., accession no. of living collection and or Specimen voucher and their respective reference.*

Supplementary Information Table S2. List of taxa used in this study with their respective GenBank accession no., accession no. of living collection and or Specimen voucher and their respective reference. — Abbreviations: BGBM= Botanical Garden and Botanical Museum Berlin-Dahlem; BGU= Botanic Gardens Utrecht; GBFM= Universidad de Panamá; GOET= Old Botanical Garden of Göttingen University; OLD= Botanical Garden of the Carl von Ossietzky University Oldenburg; REG= Botanical Garden of the University of Regensburg. — Nomenclature follows The Plant List (2013; http://www.theplantlist.org/).

Taxon	DNA Sequences				
	GenBank accession no.: (1) trnL-trnF; (2) matK		Accession no. of living collection (Botanical Garden) and or Specimen voucher (Voucher deposition)		Reference
	(1)	(2)	(1)	(2)	
**Bromeliaceae subf. Bromelioideae**					
Aechmea angustifolia Poepp. & Endl.	KU702787	KU702732	14-098-06-90 (OLD)		(1) Müller *et al.* 2017; (2) Müller *et al.* 2017
Aechmea aquilega (Salisb.) Griseb.	FJ942886	JN202203	—		(1) Sass & Specht 2010; (2) Maia *et al.* 2012
Aechmea bracteata (Sw.) Griseb.	KU702788	KU702733	00981 (OLD)		(1) Müller *et al.* 2017; (2) Müller *et al.* 2017
Aechmea coelestis (K.Koch) E.Morren	KU702789	KU702734	1997GR00639 (BGU)		(1) Müller *et al.* 2017; (2) Müller *et al.* 2017
Aechmea fasciata (Lindl.) Baker	FJ942896	AY950034	—		(1) Sass & Specht 2010; (2) Schulte *et al.* 2005
Aechmea filicaulis (Griseb.) Mez	KU702790	KU702735	14-098-09-90 (OLD)		(1) Müller *et al.* 2017; (2) Müller *et al.* 2017
Aechmea fosteriana L.B.Sm.	KU762945	KU762888	—		(1) Aguirre-Santoro *et al.* 2016; (2) Aguirre-Santoro *et al.* 2016
Aechmea melinonii Hook.	KU702791	KU702736	093-02-08-74 (BGBM)		(1) Müller *et al.* 2017; (2) Müller *et al.* 2017
Aechmea mexicana Baker	KU702792	KU702737	00961 (OLD)		(1) Müller *et al.* 2017; (2) Müller *et al.* 2017
Aechmea nudicaulis (L.) Griseb.	AY614268	AY614024	—		(1) Barfuss *et al.* 2005; (2) Barfuss *et al.* 2005
Aechmea racinae L.B.Sm	KU702793	KU702738	013-17-91-33 (BGBM)		(1) Müller *et al.* 2017; (2) Müller *et al.* 2017
Aechmea ramosa Mart. ex Schult. & Schult.f.	KJ580301	JN202231	—		(1) Evans *et al.* 2015; (2) Maia *et al.* 2012

Taxon	DNA Sequences				
	GenBank accession no.: (1) trnL-trnF; (2) matK		Accession no. of living collection (Botanical Garden) and or Specimen voucher (Voucher deposition)		Reference
	(1)	(2)	(1)	(2)	
**Bromeliaceae subf. Bromelioideae continue**					
Aechmea veitchii Baker	KU702794	KU702739	00942 (OLD)		(1) Müller *et al.* 2017; (2) Müller *et al.* 2017
Aechmea victoriana L.B.Sm.	KU702795	EF110633	14-098-07-90 (OLD)	—	(1) Müller *et al.* 2017; (2) de Sousa *et al.* 2007
	KU702795	KU702740	14-098-07-90 (OLD)		(1) Müller *et al.* 2017; (2) Müller *et al.* 2017
Aechmea weberbaueri Harms	KU702796	KU702741	2012M3 (750) (REG)		(1) Müller *et al.* 2017; (2) Müller *et al.* 2017
Aechmea weilbachii Didr.	KU702797	KU702742	14-098-12-90 (OLD)		(1) Müller *et al.* 2017; (2) Müller *et al.* 2017
Aechmea wuelfinghoffii E.Gross	KU702798	KU702743	14-098-13-90 (OLD)		(1) Müller *et al.* 2017; (2) Müller *et al.* 2017
Ananas bracteatus (Lindl.) Schult. & Schult.f.	JX649281	JX649241	—		(1) Silvestro *et al.* unpubl.; (2) Silvestro *et al.* unpubl.
Ananas comosus (L.) Merr.	KU762973	KJ580030	—		(1) Aguirre-Santoro *et al.* 2016; (2) Evans *et al.* 2015
Billbergia amoena (Lodd.) Lindl.	KU702799	JX649243	14-098-14-90 (OLD)	—	(1) Müller *et al.* 2017; (2) Silvestro *et al.* unpubl.
	KU702799	KU702744	14-098-14-90 (OLD)		(1) Müller *et al.* 2017; (2) Müller *et al.* 2017
Billbergia brasiliensis L.B.Sm.	KU702800	KU702745	14-098-16-90 (OLD)		(1) Müller *et al.* 2017; (2) Müller *et al.* 2017
Billbergia chlorantha L.B.Sm.	KU702801	KU702746	1969/0386 (REG)		(1) Müller *et al.* 2017; (2) Müller *et al.* 2017
Billbergia horrida Regel	KJ580333	KJ580041	—		(1) Evans *et al.* 2015; (2) Evans *et al.* 2015
Billbergia magnifica Mez	KU702802	KU702747	14-098-17-90 (OLD)		(1) Müller *et al.* 2017; (2) Müller *et al.* 2017
Billbergia nutans H.Wendl. ex Regel	KJ580339	KJ580047	—		(1) Evans *et al.* 2015; (2) Evans *et al.* 2015
Billbergia pallidiflora Liebm.	FJ942949	KU762819	—		(1) Sass & Specht 2010; (2) Aguirre-Santoro *et al.* 2016
Billbergia pyramidalis (Sims) Lindl.	FJ942950	AY952422	—		(1) Sass & Specht 2010; (2) Li & Zhou unpubl.
Billbergia tweedieana Baker	JX649297	JX649244	—		(1) Silvestro *et al.* unpubl.; (2) Silvestro *et al.* unpubl.
Billbergia velascana Cárdenas	KU702803	KU702748	14-098-19-90 (OLD)		(1) Müller *et al.* 2017; (2) Müller *et al.* 2017

Taxon	DNA Sequences				
	GenBank accession no.: (1) trnL-trnF; (2) matK		Accession no. of living collection (Botanical Garden) and or Specimen voucher (Voucher deposition)		Reference
	(1)	(2)	(1)	(2)	
**Bromeliaceae subf. Bromelioideae continue**					
Billbergia viridiflora H.Wendl.	FJ942952	KU762805	—		(1) Sass & Specht 2010; (2) Aguirre-Santoro *et al.* 2016
Canistrum fragrans (Linden) Mabb.	DQ084631	AY950012	—		(1) Horres *et al.* 2007; (2) Schulte *et al.* 2005
Cryptanthus bahianus L.B.Sm.	KJ676905	AY950011	—		(1) Louzada *et al.* 2014; (2) Schulte *et al.* 2005
Cryptanthus beuckeri E.Morren	HQ882723	AF539965	—		(1) Givnish *et al.* 2011; (2) Crayn *et al.* 2004
Neoregelia carolinae (Beer) L.B.Sm.	KU702804	HQ180877	14-098-30-90 (OLD)	—	(1) Müller *et al.* 2017; (2) Givnish *et al.* 2010
	KU702804	KU702749	14-098-30-90 (OLD)		(1) Müller *et al.* 2017; (2) Müller *et al.* 2017
Neoregelia zonata L.B.Sm.	KU702805	KU702750	14-098-32-90 (OLD)		(1) Müller *et al.* 2017; (2) Müller *et al.* 2017
Nidularium billbergioides (Schult. & Schult.f.) L.B.Sm.	KU702806	KU702751	2000/3126 (REG)		(1) Müller *et al.* 2017; (2) Müller *et al.* 2017
Nidularium campos-portoi (L.B.Sm.) Leme	KU702807	KU702752	14-098-60-90 (OLD)		(1) Müller *et al.* 2017; (2) Müller *et al.* 2017
Nidularium innocentii Lem.	KU702804	JN202259	14-098-31-90(OLD)	—	(1) Müller *et al.* 2017; (2) Maia *et al.* 2012
	KU702804	KU702749	14-098-31-90 (OLD)		(1) Müller *et al.* 2017; (2) Müller *et al.* 2017
Ochagavia elegans Phil.	KJ580390	FJ968167	—		(1) Evans *et al.* 2015; (2) Jabaily & Sytsma 2010
Orthophytum foliosum L.B.Sm.	KU702809	KU702754	336-13-96-84 (BGBM)		(1) Müller *et al.* 2017; (2) Müller *et al.* 2017
Orthophytum saxicola (Ule) L.B.Sm.	KJ676947	JX649269	—		(1) Louzada *et al.* 2014; (2) Silvestro *et al.* unpubl.
Ronnbergia deleonii L.B.Sm.	KU702810	KU702755	14-098-35-90 (OLD)		(1) Müller *et al.* 2017; (2) Müller *et al.* 2017
**Bromeliaceae subf. Tillandsioideae**					
Catopsis juncifolia Mez & Wercklé	AY614271	AY614027	—		(1) Barfuss *et al.* 2005; (2) Barfuss *et al.* 2005
Catopsis morreniana Mez	AY614269	AY614025	—		(1) Barfuss *et al.* 2005; (2) Barfuss *et al.* 2005
Catopsis nitida (Hook.) Griseb.	—	KX754166	—		(1) Barfuss, M. unpubl.; (2) Barfuss *et al.* 2016

Taxon	DNA Sequences				
	GenBank accession no.: (1) trnL-trnF; (2) matK		Accession no. of living collection (Botanical Garden) and or Specimen voucher (Voucher deposition)		Reference
	(1)	(2)	(1)	(2)	
**Bromeliaceae subf. Tillandsioideae continue**					
Catopsis sessiliflora (Ruiz & Pav.) Mez	KU702811	—	LL. Müller s.n.(GBFM)	—	(1) Müller *et al.* 2017; (2) Barfuss, M. unpubl.
	KU702811	—	LL. Müller s.n.(GBFM)	—	(1) Müller *et al.* 2017; (2) Barfuss, M. unpubl.
Catopsis subulata L.B.Sm.	AY614272	AY614028	—		(1) Barfuss *et al.* 2005; (2) Barfuss *et al.* 2005
Guzmania acorifolia (Griseb.) Mez	AY614304	AY614060	—		(1) Barfuss *et al.* 2005; (2) Barfuss *et al.* 2005
Guzmania butcheri Rauh	KU702812	KU702756	179-21-11-80 (BGBM)		(1) Müller *et al.* 2017; (2) Müller *et al.* 2017
Guzmania lingulata (L.) Mez	KU702813	KU702757	156-05-97-34 (BGBM)		(1) Müller *et al.* 2017; (2) Müller *et al.* 2017
Guzmania monostachia (L.) Rusby ex Mez	HQ882732	AF162243	—		(1) Givnish *et al.* 2011; (2) Crayn *et al.* 2004
Guzmania osyana (E.Morren) Mez	KU702814	KU702758	14-127-04-90 (OLD)		(1) Müller *et al.* 2017; (2) Müller *et al.* 2017
Guzmania patula Mez & Wercklé	AY614309	AY614065	—		(1) Barfuss *et al.* 2005; (2) Barfuss *et al.* 2005
	AY614309	AY614065	—		(1) Barfuss *et al.* 2005; (2) Barfuss *et al.* 2005
Guzmania subcorymbosa L.B.Sm.	KU702815	JQ587152	253-19-92-84 (BGBM)	—	(1) Müller *et al.* 2017; (2) iBOL Data unpubl.
Guzmania wittmackii (André) André ex Mez	AY614300	AY949991	—		(1) Barfuss *et al.* 2005; (2) Schulte *et al.* 2005
Guzmania zahnii (Hook.f.) Mez	KU702816	KU702759	14-098-28-90 (OLD)		(1) Müller *et al.* 2017; (2) Müller *et al.* 2017
Tillandsia anceps Lodd.	—	—	—		(1) Barfuss, M. unpubl.; (2) Barfuss, M. unpubl.
	—	—	—		(1) Barfuss, M. unpubl.; (2) Barfuss, M. unpubl.
Tillandsia andicola Gillies ex Baker	KU702817	KU702760	263-24-97-63 (BGBM)		(1) Müller *et al.* 2017; (2) Müller *et al.* 2017
Tillandsia baileyi Rose ex Small	—	—	—		(1) Barfuss, M. unpubl.; (2) Barfuss, M. unpubl.
	—	—	—		(1) Barfuss, M. unpubl.; (2) Barfuss, M. unpubl.
Tillandsia balbisiana Schult. & Schult.f.	KU702818	KU702761	GT-0-GOET-20039 (GOET)		(1) Müller *et al.* 2017; (2) Müller *et al.* 2017
Tillandsia bulbosa Hook.	KU702819	JN202272	G. Zotz s.n. (GBFM)	—	(1) Müller *et al.* 2017; (2) Maia *et al.* 2012

Taxon	DNA Sequences				
	GenBank accession no.: (1) trnL-trnF; (2) matK		Accession no. of living collection (Botanical Garden) and or Specimen voucher (Voucher deposition)		Reference
	(1)	(2)	(1)	(2)	
**Bromeliaceae subf. Tillandsioideae continue**					
Tillandsia caput-medusae E.Morren	AY614342	AY614098	—		(1) Barfuss *et al.* 2005; (2) Barfuss *et al.* 2005
Tillandsia cyanea Linden ex K.Koch	—	—	—		(1) Barfuss, M. unpubl.; (2) Barfuss, M. unpubl.
	—	—	—		(1) Barfuss, M. unpubl.; (2) Barfuss, M. unpubl.
Tillandsia didisticha (E.Morren) Baker	AY614371	AY614127	—		(1) Barfuss *et al.* 2005; (2) Barfuss *et al.* 2005
Tillandsia elongata Kunth	KU702820	KU702762	G. Zotz s.n. (GBFM)		(1) Müller *et al.* 2017; (2) Müller *et al.* 2017
Tillandsia fasciculata Sw.	FM211666	FM210792	—		(1) de Castro *et al.* 2009; (2) de Castro *et al.* 2009
Tillandsia flabellata Baker	KU702842	KU702763	14-098-40-90 (OLD)		(1) Müller *et al.* 2017; (2) Müller *et al.* 2017
Tillandsia flexuosa Sw.	KU702843	KU702764	Zotz s.n. (GBFM)		(1) Müller *et al.* 2017; (2) Müller *et al.* 2017
Tillandsia fraseri Baker	—	AF539977	—		(1) Barfuss, M. unpubl.; (2) Crayn *et al.* 2004
	—	—	—		(1) Barfuss, M. unpubl.; (2) Barfuss, M. unpubl.
	—	KX754176	—		(1) Barfuss, M. unpubl.; (2) Barfuss *et al.* 2016
	—	—	—		(1) Barfuss, M. unpubl.; (2) Barfuss, M. unpubl.
	—	—	—		(1) Barfuss, M. unpubl.; (2) Barfuss, M. unpubl.
Tillandsia funckiana Baker	KU702821	KU702765	14-098-41-90 (OLD)		(1) Müller *et al.* 2017; (2) Müller *et al.* 2017
Tillandsia heliconioides (Kunth)	KU702822	KU702766	2013GR00767 (BGU)		(1) Müller *et al.* 2017; (2) Müller *et al.* 2017
Tillandsia heterophylla E.Morren	AY614355	AY614111	—		(1) Barfuss *et al.* 2005; (2) Barfuss *et al.* 2005
Tillandsia ionantha Planch.	KU702823	KU702767	00940 (OLD)		(1) Müller *et al.* 2017; (2) Müller *et al.* 2017
Tillandsia juncea (Ruiz & Pav.) Poir.	AY614341	AY614097	—		(1) Barfuss *et al.* 2005; (2) Barfuss *et al.* 2005
Tillandsia landbeckii ssp. *andina* Phil.	—	KX754162	—		(1) Barfuss, M. unpubl.; (2) Barfuss *et al.* 2016
Tillandsia latifolia Meyen	AY614352	AY614108	—		(1) Barfuss *et al.* 2005; (2) Barfuss *et al.* 2005

Taxon	DNA Sequences				
	GenBank accession no.: (1) trnL-trnF; (2) matK		Accession no. of living collection (Botanical Garden) and or Specimen voucher (Voucher deposition)		Reference
	(1)	(2)	(1)	(2)	
**Bromeliaceae subf. Tillandsioideae continue**					
Tillandsia monadelpha (E.Morren) Baker	KU702824	KU702768	Zotz s.n. (GBFM)		(1) Müller *et al.* 2017; (2) Müller *et al.* 2017
Tillandsia narthecioides C.Presl	AY614315	AY614071	—		(1) Barfuss *et al.* 2005; (2) Barfuss *et al.* 2005
Tillandsia oerstediana L.B.Sm.	KU702825	KU702769	00989 (OLD)		(1) Müller *et al.* 2017; (2) Müller *et al.* 2017
Tillandsia pucaraensis Ehlers	KU702826	KU702770	(OLD)		(1) Müller *et al.* 2017; (2) Müller *et al.* 2017
Tillandsia punctulata Schltdl. & Cham.	AY614331	AY614087	—		(1) Barfuss *et al.* 2005; (2) Barfuss *et al.* 2005
Tillandsia rauhii L.B.Sm.	AY614345	AY614101	—		(1) Barfuss *et al.* 2005; (2) Barfuss *et al.* 2005
Tillandsia recurvata (Gaudich.) Baker	KU702827	KU702771	Zotz s.n. (GBFM)		(1) Müller *et al.* 2017; (2) Müller *et al.* 2017
Tillandsia remota Wittm.	AY614339	AY614095	—		(1) Barfuss *et al.* 2005; (2) Barfuss *et al.* 2005
Tillandsia ropalocarpa André	AY614327	AY614083	—		(1) Barfuss *et al.* 2005; (2) Barfuss *et al.* 2005
Tillandsia stricta Sol. ex Ker Gawl.	AY614374	AY614130	—		(1) Barfuss *et al.* 2005; (2) Barfuss *et al.* 2005
Tillandsia subulifera Mez	KU702828	KU702772	Zotz s.n. (GBFM)		(1) Müller *et al.* 2017; (2) Müller *et al.* 2017
Tillandsia tectorum E.Morren	AY614317	AY614073	—		(1) Barfuss *et al.* 2005; (2) Barfuss *et al.* 2005
Tillandsia usneoides (L.) L.	AY614365	AY614121	—		(1) Barfuss *et al.* 2005; (2) Barfuss *et al.* 2005
Tillandsia viridiflora (Beer) Baker	AY614310	AY614066	—		(1) Barfuss *et al.* 2005; (2) Barfuss *et al.* 2005
Vriesea bicolor L.B.Sm.	KU702829	KU702773	Zotz s.n. (GBFM)		(1) Müller *et al.* 2017; (2) Müller *et al.* 2017
Vriesea bituminosa Wawra	AY614282	AY614038	—		(1) Barfuss *et al.* 2005; (2) Barfuss *et al.* 2005
Vriesea bleheri Roeth & W.Weber	KU702830	KU702774	14-098-50-90 (OLD)		(1) Müller *et al.* 2017; (2) Müller *et al.* 2017
Vriesea burgeri L.B.Sm.	KU702831	KU702775	Zotz s.n. (GBFM)		(1) Müller *et al.* 2017; (2) Müller *et al.* 2017
Vriesea carinata Wawra	AY614277	AY614033	—		(1) Barfuss *et al.* 2005; (2) Barfuss *et al.* 2005
Vriesea dubia (L.B.Sm.) L.B.Sm.	KU702832	KU702776	14-098-49-90 (OLD)		(1) Müller *et al.* 2017; (2) Müller *et al.* 2017

Taxon	DNA Sequences				
	GenBank accession no.: (1) trnL-trnF; (2) matK		Accession no. of living collection (Botanical Garden) and or Specimen voucher (Voucher deposition)		Reference
	(1)	(2)	(1)	(2)	
**Bromeliaceae subf. Tillandsioideae continue**					
Vriesea dubia (L.B.Sm.) L.B.Sm.	KU702832	KU702776	14-098-49-90 (OLD)		(1) Müller *et al.* 2017; (2) Müller *et al.* 2017
Vriesea duvaliana E.Morren	KU702833	KU702777	14-098-51-90 (OLD)		(1) Müller *et al.* 2017; (2) Müller *et al.* 2017
	KU702844	KU702786	14-098-51-1-90 (OLD)		(1) Müller *et al.* 2017; (2) Müller *et al.* 2017
Vriesea fenestralis Linden & André	KU702834	KU702778	14-098-52-90 (OLD)		(1) Müller *et al.* 2017; (2) Müller *et al.* 2017
Vriesea geniculata (Wawra) Wawra	KU702835	KU702779	2007GR01394 (BGU)		(1) Müller *et al.* 2017; (2) Müller *et al.* 2017
	KU702836	KU702780	289-87-00-30 (BGBM)		(1) Müller *et al.* 2017; (2) Müller *et al.* 2017
Vriesea gladioliflora (H.Wendl.) Antoine	—	KX754116	—		(1) Barfuss, M. unpubl.; (2) Barfuss *et al.* 2016
	—	—	—		(1) Barfuss, M. unpubl.; (2) Barfuss, M. unpubl.
	—	—	—		(1) Barfuss, M. unpubl.; (2) Barfuss, M. unpubl.
Vriesea maxoniana (L.B.Sm.) L.B.Sm.	—	—	—		(1) Barfuss, M. unpubl.; (2) Barfuss, M. unpubl.
	—	KX754169	—		(1) Barfuss, M. unpubl.; (2) Barfuss *et al.* 2016
Vriesea nutans L.B.Sm.	KU702837	KU702781	14-098-58-90 (OLD)		(1) Müller *et al.* 2017; (2) Müller *et al.* 2017
Vriesea ospinae var. *gruberi* H. Luther	AY614284	AY614040	—		(1) Barfuss *et al.* 2005; (2) Barfuss *et al.* 2005
Vriesea patzeltii Rauh	KU702838	KU702782	XX-0-GOET-04293 (GOET)		(1) Müller *et al.* 2017; (2) Müller *et al.* 2017
Vriesea paupera (Mez & Sodiro) L.B.Sm. & Pittendr.	KU702839	KU702783	XX-0-GOET-04292 (GOET)		(1) Müller *et al.* 2017; (2) Müller *et al.* 2017
Vriesea sanguinolenta Cogn. & Marchal	KU702840	KU702784	00947 (OLD)		(1) Müller *et al.* 2017; (2) Müller *et al.* 2017
Vriesea splendens (Brongn.) Lem.	AY614289	AY614045	—		(1) Barfuss *et al.* 2005; (2) Barfuss *et al.* 2005
Vriesea sucrei L.B.Sm. & Read	—	—	—		(1) Barfuss, M. unpubl.; (2) Barfuss, M. unpubl.
Vriesea unilateralis (Baker) Mez	KU702841	KU702785	XX-0-GOET-00799 (GOET)		(1) Müller *et al.* 2017; (2) Müller *et al.* 2017

Taxon	DNA Sequences				
	GenBank accession no.: (1) trnL-trnF; (2) matK		Accession no. of living collection (Botanical Garden) and or Specimen voucher (Voucher deposition)		Reference
	(1)	(2)	(1)	(2)	
**Bromeliaceae subf. Tillandsioideae continue**					
Vriesea viridiflora (Regel) Wittm. ex Mez	AY614310	AY614066	—		(1) Barfuss *et al.* 2005; (2) Barfuss *et al.* 2005
Vriesea zamorensis (L.B.Sm.) L.B.Sm.	AY614287	AY614043	—		(1) Barfuss *et al.* 2005; (2) Barfuss *et al.* 2005
**Bromeliaceae subf. Brocchinioideae**					
Brocchinia acuminata L.B.Sm.	HQ882715	EU681908	—		(1) Givnish *et al.* 2011; (2) Rex *et al.* 2009
Brocchinia uaipanensis (Maguire) Givnish	HQ882717	EU681909	—		(1) Givnish *et al.* 2011; (2) Rex *et al.* 2009
Brocchinia prismatica L.B.Sm.	HQ882716	HQ900681	—		(1) Givnish *et al.* 2011; (2) Givnish *et al.* 2011

References

Aguirre-Santoro, J., Michelangeli, F.A. & Stevenson, D.W. (2016). Molecular phylogenetics of the *Ronnbergia* Alliance (Bromeliaceae, Bromelioideae) and insights into their morphological evolution. *Molecular Phylogenetics and Evolution*, **100,** 1-20.

Barfuss, M.H., Till, W., Leme, E.M., Pinzón, J.P., Manzanares, J.M., Halbritter, H. *et al.* (2016). Taxonomic revision of Bromeliaceae subfam. Tillandsioideae based on a multi-locus DNA sequence phylogeny and morphology. *Phytotaxa*, **279,** 1-97.

Barfuss, M.H.J., Samuel, R., Till, W., Stuessy, T.F., Barfuss, M.H.J., Samuel, R. *et al.* (2005). Phylogenetic relationships in subfamily Tillandsioideae (Bromeliaceae) based on DNA sequence data from seven plastid regions. *American Journal of Botany*, **92,** 337-351.

Crayn, D.M., Winter, K. & Smith, J.A.C. (2004). Multiple origins of crassulacean acid metabolism and the epiphytic habit in the Neotropical family Bromeliaceae. *Proceedings of the National Academy of Sciences of the United States of America*, **101,** 3703-3708.

de Castro, O., Cennamo, P., Vazquez-Torres, M. & de Luca, P. (2009). Molecular studies about two rare species of the genus *Tillandsia* L. (*T. califanii* Rauh and *T. tomaselii* De Luca, Sabato et Balduzzi). *Journal of the Bromeliad Society*, **59,** 206-219.

de Sousa, L.d.O.F., Wendt, T., Brown, G.K., Tuthill, D.E. & Evans, T.M. (2007). Monophyly and phylogenetic relationships in Lymania (Bromeliaceae: Bromelioideae) based on morphology and chloroplast DNA sequences. *Systematic Botany*, **32,** 264-270.

Evans, T.M., Jabaily, R.S., de Faria, A.P.G., de Sousa, L.d.O.F., Wendt, T. & Brown, G.K. (2015). Phylogenetic relationships in Bromeliaceae subfamily Bromelioideae based on chloroplast DNA sequence data. *Systematic Botany*, **40,** 116-128.

Givnish, T.J., Ames, M., McNeal, J.R., McKain, M.R., Steele, P.R., Graham, S.W. *et al.* (2010). Assembling the tree of the monocotyledons: plastome sequence phylogeny and evolution of poales. *Annals of the Missouri Botanical Garden*, **97,** 584-616.

Givnish, T.J., Barfuss, M.H.J., van Ee, B., Riina, R., Schulte, K., Horres, R. *et al.* (2011). Phylogeny, adaptive radiation, and historical biogeography in Bromeliaceae: insights from an eight-locus plastid phylogeny. *American Journal of Botany*, **98,** 872-895.

Horres, R., Schulte, K., Weising, K. & Zizka, G. (2007). Systematics of Bromelioideae (Bromeliaceae)—evidence from molecular and anatomical studies. *Aliso: A Journal of Systematic and Evolutionary Botany*, **23,** 27-43.

Jabaily, R.S. & Sytsma, K.J. (2010). Phylogenetics of *Puya* (Bromeliaceae): placement, major lineages, and evolution of Chilean species. *American Journal of Botany*, **97,** 337-356.

Louzada, R.B., Schulte, K., Wanderley, M.G.L., Silvestro, D., Zizka, G., Barfuss, M.H. *et al.* (2014). Molecular phylogeny of the Brazilian endemic genus *Orthophytum* (Bromelioideae, Bromeliaceae) and its implications on morphological character evolution. *Molecular Phylogenetics and Evolution*, **77,** 54-64.

Maia, V.H., Da Mata, C.S., Franco, L.O., Cardoso, M.A., Cardoso, S.R.S., Hemerly, A.S. *et al.* (2012). DNA barcoding Bromeliaceae: achievements and pitfalls. *PLoS ONE*, **7,** e29877.

Müller, L.-L.B., Albach, D.C., & Zotz, G. (2017). "Are 3°C too much?": thermal niche breadth in Bromeliaceae and global warming. *Journal of Ecology*, **105,** 507-516.

Rex, M., Schulte, K., Zizka, G., Peters, J., Vásquez, R., Ibisch, P.L. *et al.* (2009). Phylogenetic analysis of *Fosterella* LB Sm.(Pitcairnioideae, Bromeliaceae) based on four chloroplast DNA regions. *Molecular Phylogenetics and Evolution*, **51,** 472-485.

Sass, C. & Specht, C.D. (2010). Phylogenetic estimation of the core Bromelioids with an emphasis on the genus *Aechmea* (Bromeliaceae). *Molecular Phylogenetics and Evolution*, **55,** 559-571.

Schulte, K., Horres, R. & Zizka, G. (2005). Molecular phylogeny of Bromelioideae and its implications on biogeography and the evolution of CAM in the family. *Senckenbergiana biologica*, **85,** 1-13.

Table S3 Results of the regression analyses across all bromeliad species and for Bromelioideae and Tillandsioideae analysed individually for genome size with relative growth rate and three growth components, respectively, without excluding polyploids.

Supplementary Information Table S3. Results of the regression analyses without putative the polyploids *Orthophytum foliosum*, *Tillandsia flexuosa* and *T. bailyi* across all bromeliad species (n = 13) and for Bromelioideae (n = 6) and Tillandsioideae (n = 7) analysed individually for genome size (2*C* DNA content) with relative growth rate (*RGR*) and with the growth components net assimilation rate (*NAR*), leaf area ratio (*LAR*) and specific leaf area (*SLA*).

	RGR (mg g^{-1} day^{-1})			*NAR* (g m^{-2} day^{-1})		
	R^2	*Slope*	*P*	R^2	*Slope*	*P*
All 2*C* DNA content (pg)	−0.05	-0.29	0.55	−0.03	-0.37	0.43
Bromelioideae 2*C* DNA content (pg)	0.08	0.95	0.29	0.73	0.73	**0.02**
Tillandsioideae 2*C* DNA content (pg)	0.77	0.95	**0.01**	0.45	1.08	0.06
	LAR (cm^2 g^{-1})			*SLA* (m^2 kg^{-1})		
	R^2	Slope	*P*	R^2	Slope	*P*
All 2*C* DNA content (pg)	-0.04	−53.43	0.49	0.29	−0.78	**0.03**
Bromelioideae 2*C* DNA content (pg)	−0.23	−51.05	0.81	−0.11	−0.51	0.52
Tillandsioideae 2*C* DNA content (pg)	−0.17	−47.06	0.72	-0.14	−0.25	0.64

Significant relationships (*P*<0.05) are indicated in bold.

Figure S1 *Phylogenetic tree of combined cpDNA dataset for 133 taxa of Bromeliaceae (Bromelioideae and Tillandsioideae) based on maximum likelihood.*

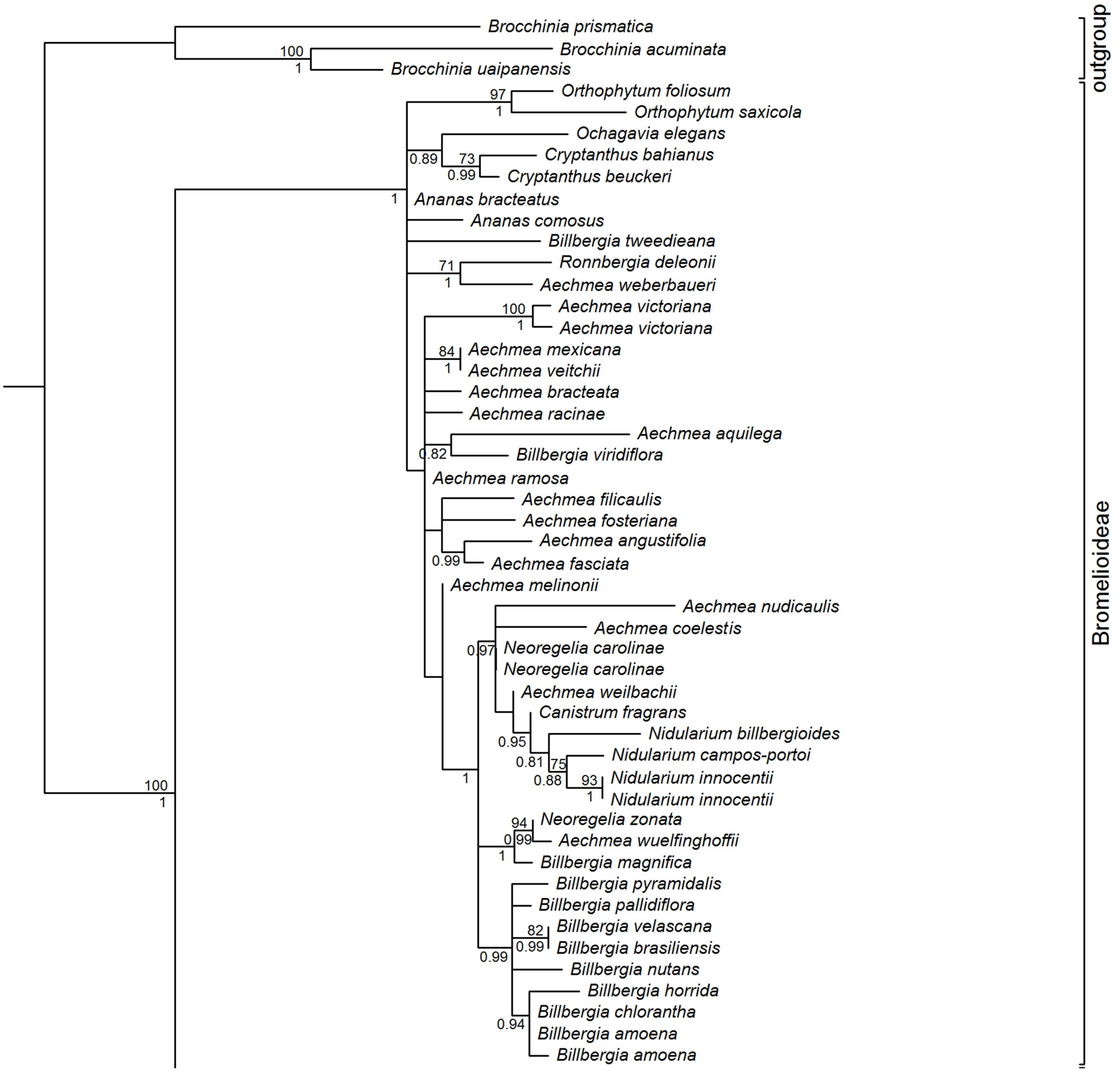

▲►**Supplementary Information Figure S1.** Phylogenetic tree of combined cpDNA dataset (*mat*K and *trn*L-F) for 133 taxa of Bromeliaceae (Bromelioideae and Tillandsioideae) based on maximum likelihood. The tree is rooted by defining *Brocchinia* as outgroup. Bootstrap support values (>70%) are plotted above each relevant node, while Bayesian posterior probabilities (>0.8) are plotted below each relevant node.

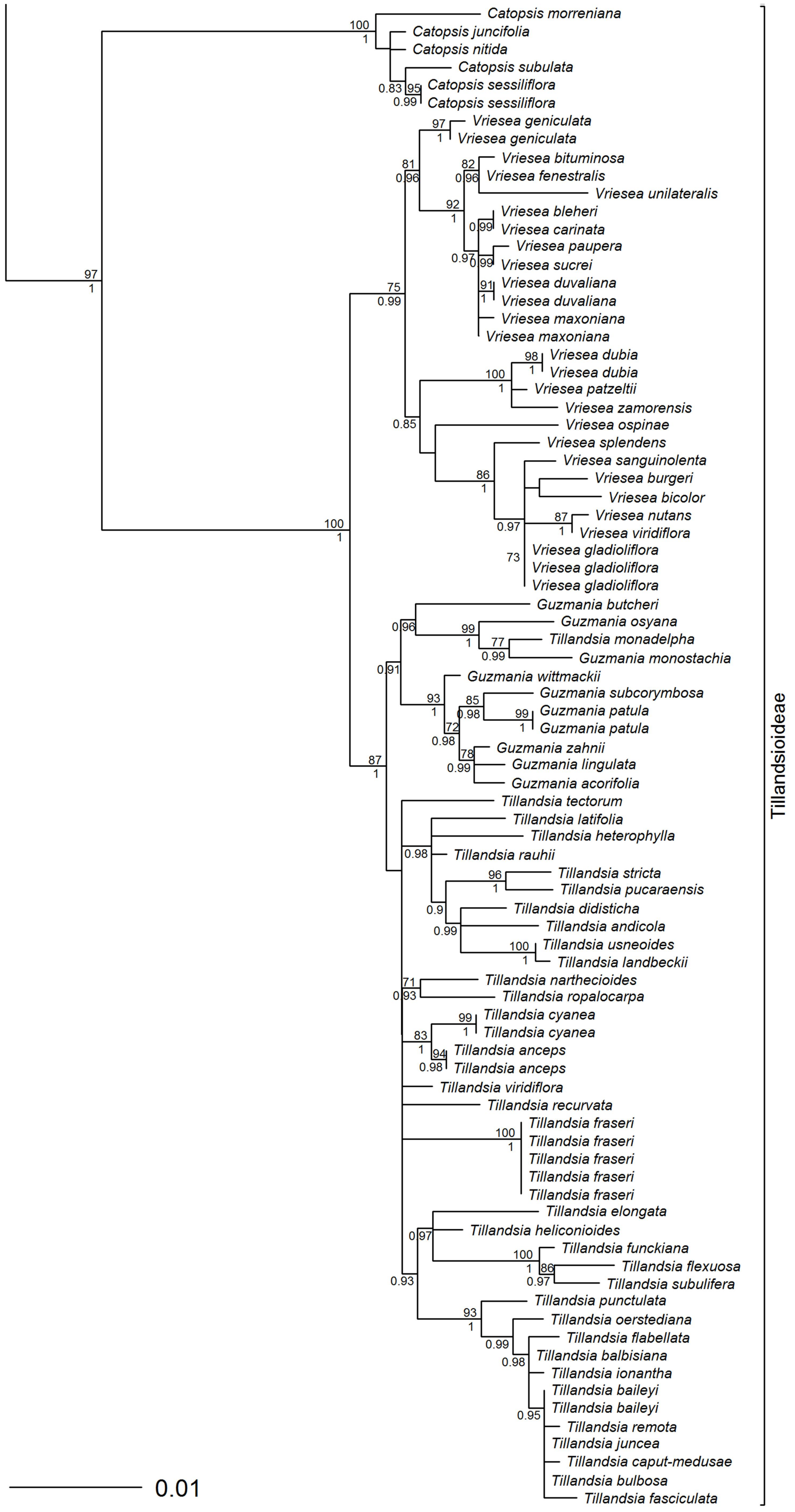

▲Supplementary Information Figure S1 continued.

Figure S2 *Pruned phylogenetic tree of combined cpDNA dataset for 105 bromeliad species with respective genome size value shown as color*

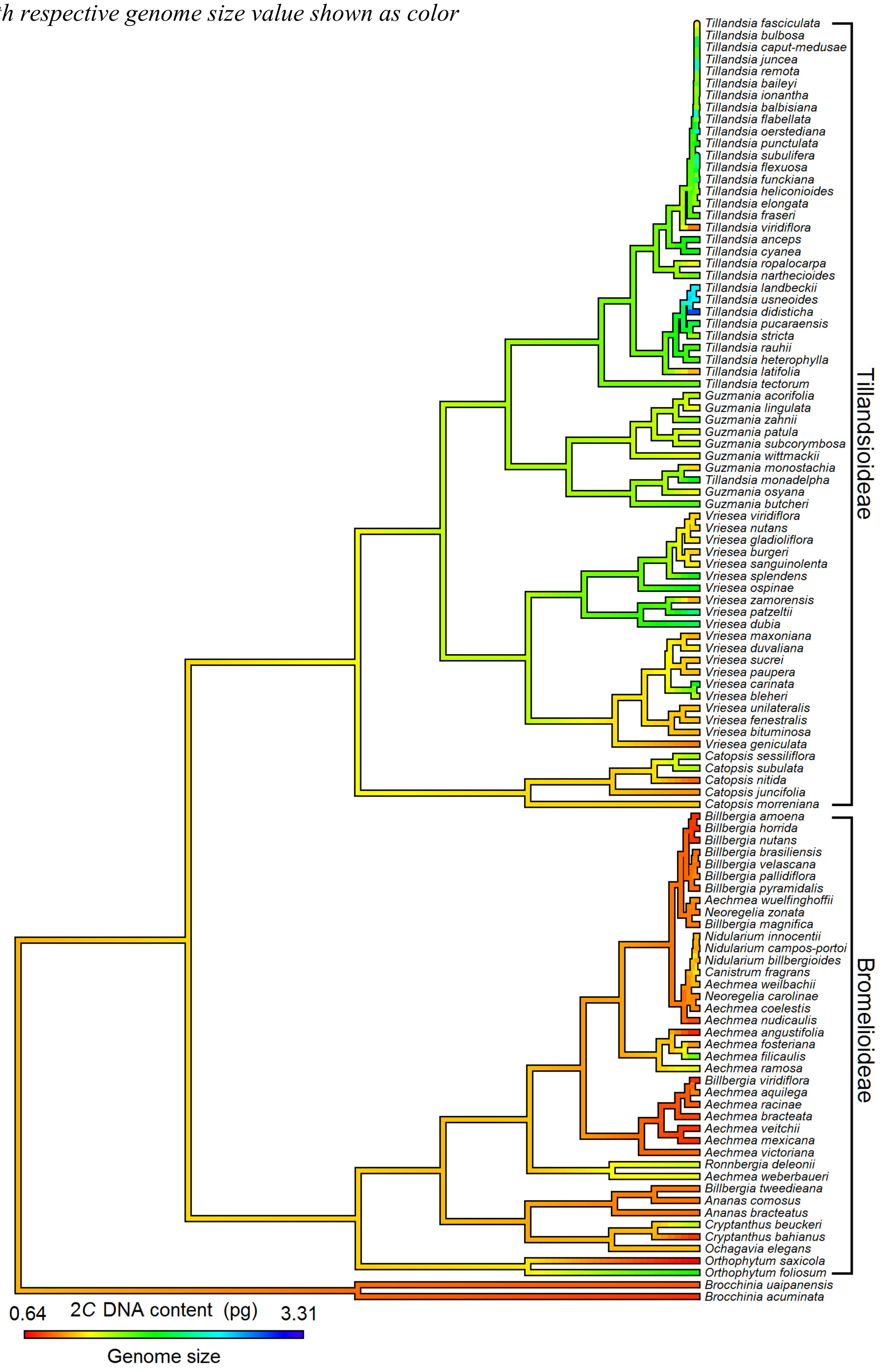

◄**Supplementary Information Figure S2.** Pruned phylogenetic tree of combined cpDNA dataset (*mat*K, *trn*L-F) of 105 bromeliad species of the subfamilies Tillandsioideae and Bromelioideae based on maximum likelihood. *Brocchinia uaipanensis* and *Brocchinia acuminata* are out-groups. Branch colors indicate ancestral and respective genome size (2*C* DNA content) values, calculated by fastAnc in the R package PHYTOOLS, ranging from small in red to large in blue. Since we consider ancestral genome size estimations speculative the further back in time we go based on the large impact of species-specific effects on genome size evolution (large values of Pagel´s δ), we do not comment on the ancestral values.

►**Supplementary Information Figure S3.** Distribution of genome size among 107 bromeliad species, sorted by tip number. Tip number based on phylogenetic tree of combined cpDNA dataset (Fig. 2). Colors indicate respective subfamily. Species names in pink indicate species used in the regression analyses.

Figure S3 *Distribution of genome size (2C DNA content) among 107 bromeliad species*

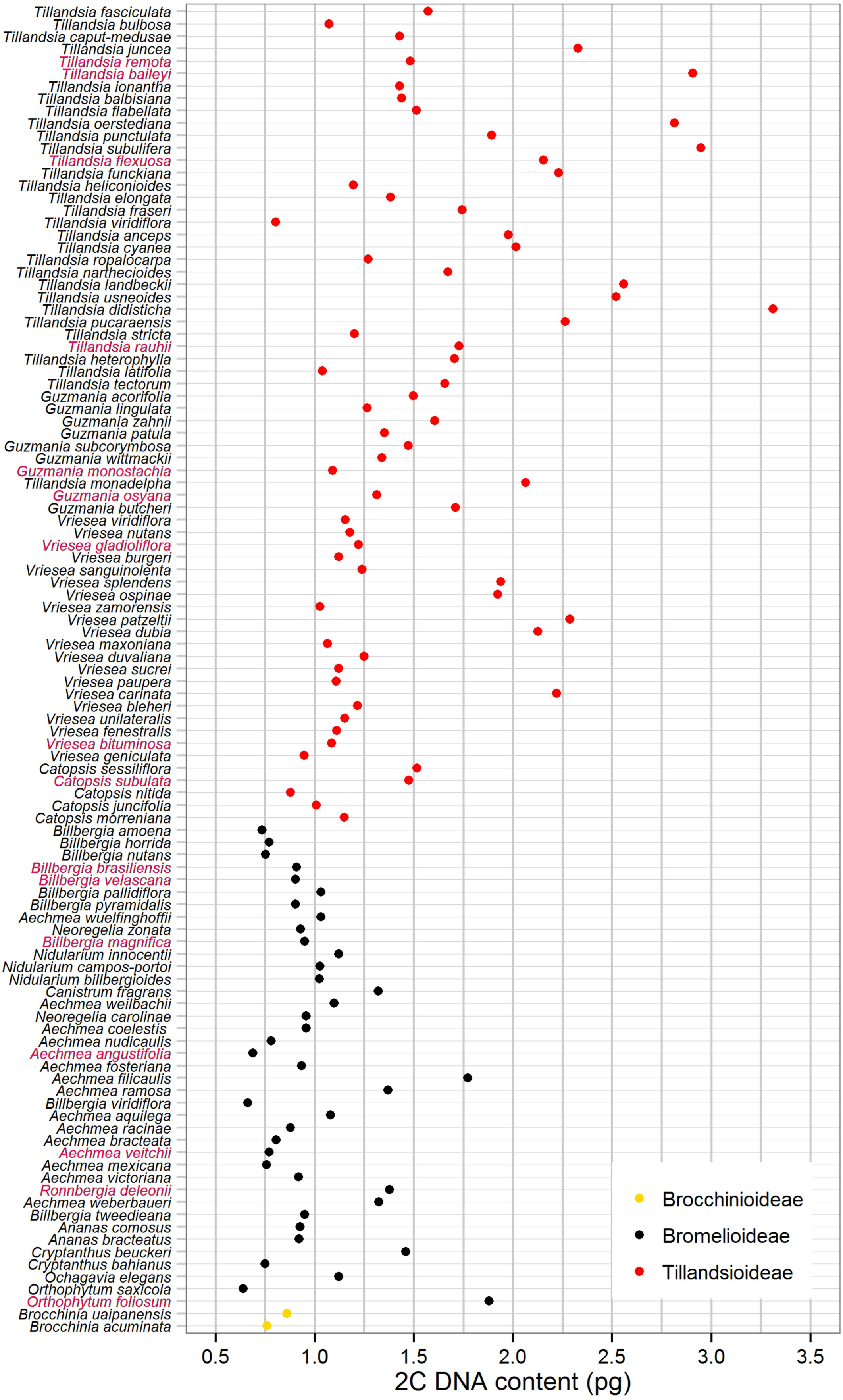

Figure S4 Relationship between genome size (2*C* DNA content) and relative growth rate and three growth components, respectively, excluding putative polyploids

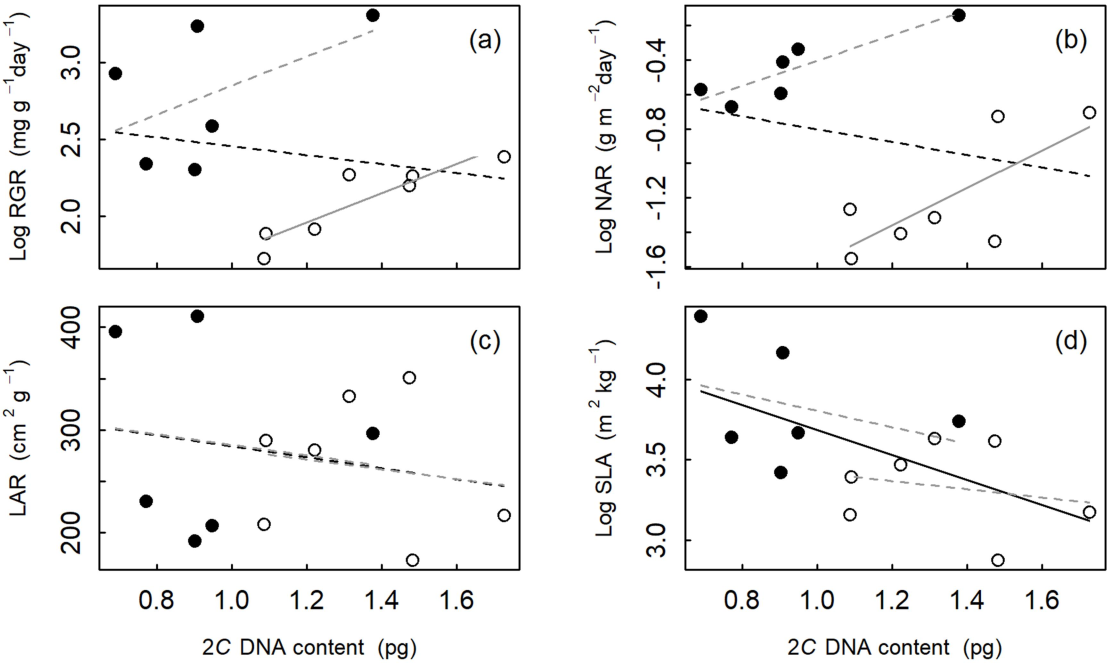

▲**Supplementary Information Figure S4.** Relationship between genome size (2*C* DNA content) and relative growth rate (*RGR*) and three growth components, respectively, across all bromeliad species (black) and across Bromelioideae and Tillandsioideae separately (grey) without the putative polyploids *Orthophytum foliosum*, *Tillandsia flexuosa* and *T. bailyi*; (a) *RGR*; (b) net assimilation rate (*NAR*); (c) leaf area ratio (*LAR*) and (d) specific leaf area (*SLA*). Data are split into Bromelioideae (closed circles) and Tillandsioideae (open circles). Solid and dashed regression lines indicate a significant and non-significant relationship, respectively.
